# Synchronous surface electromyography as objective method to evaluate the outcome of a biofeedback training in patients with facial synkinesis

**DOI:** 10.1038/s41598-025-01278-7

**Published:** 2025-05-19

**Authors:** Richard Schneider, Maren Schramm, Paul F. Funk, Gerd Fabian Volk, Christoph Anders, Orlando Guntinas-Lichius

**Affiliations:** 1 Department of Otolaryngology, Jena University Hospital, Friedrich-Schiller-University Jena, Am Klinikum 1, 07747 Jena, Germany; 2 Division Motor Research, Pathophysiology and Biomechanics, Department of Trauma, Hand and Reconstructive Surgery, Jena University Hospital, Friedrich-Schiller-University Jena, Jena, Germany; 3 Facial Nerve Center Jena, Jena University Hospital, Jena, Germany; 4 Center for Rare Diseases, Jena University Hospital, Jena, Germany

**Keywords:** Neurological disorders, Neuromuscular disease

## Abstract

Facial aberrant reinnervation after unilateral facial paralysis is characterized by facial synkinesis and global facial muscle hypertonicity. Therefore, therapy effort is directed on improved facial symmetry by reducing facial synkinesis and the elevated muscle tone. There are no established methods to confirm these aims objectively. Therefore the aim of the present study was to verify if high-resolution surface electromyography (HR-sEMG) mapping of the entire face during standardized facial movements is one such sought-after method. Bilateral HR-sEMG facial mapping was performed in 36 patients (81% women; age range: 24–70 years) with a postparalytic facial nerve syndrome. Participants performed a standard set of standardized facial movement tasks before start (T0) and after nine days of training (T9). A linear mixed-effects model was used to evaluate differences between the facial movement tasks in-between the synkinetic side and the contralateral side at T0 and T9. The overall facial muscle activity was higher on the synkinetic side compared to the contralateral side at T0 (*p* < 0.001) and also at T9, but with reduced difference between sides (*p* ≤ 0.002). The overall muscle activity decreased on the synkinetic side and on the contralateral side (both *p* < 0.001). These effects were also verifiable for almost every investigated muscle. HR-sEMG facial mapping proved its suitability as an objective method to confirm facial feedback training effects: A combined visual and EMG-based facial biofeedback training seemed to reduce the facial muscle activity on both facial sides, but markedly more effective on the synkinetic side.

**Supplementary Information:**

The online version contains supplementary material available at 10.1038/s41598-025-01278-7.

## Introduction

Severe facial nerve lesion with axonal damage and spontaneous nerve re-sprouting or induced nerve re-growth by nerve reconstruction surgery leads to misguided aberrant nerve regeneration. An important clinical sequela is the postparalytic facial nerve syndrome with synkinesis, shortly often called facial synkinesis. Facial synkinesis is characterized by unintentional facial muscle activation together with unmeant mimic movement occurring simultaneously with intentional facial movements^1,2^. A typical complaint is involuntary eye closure during intentional mouth movements while speaking or eating which is considered a significant disturbing outcome. Another, less frequently mentioned yet equally significant consequence is the increased resting tone of facial muscles, known as facial muscular hypertonicity^3^. The reasons of this hypertonicity are not fully understood. One reason could be muscle hyperinnervation. It occurs when aberrant regeneration leads to an excessive number of nerve fibers re-supplying a facial muscle. Furthermore, when facial muscles are frequently activated in an unintentional manner due to synkinetic activity, they tend to develop increased tone and stiffness over time. These continuous contractions can prevent the muscles from fully relaxing, leading to a hypertonic state. A deepened nasolabial fold, decreased palpebral fissure, or retracted angle of the mouth may indicate increased resting tone. Patients commonly experience facial muscle fatigue as a result of the elevated resting tension.

Facial synkinesis and facial muscle hypertonicity are not only leading to functional deficits. The patients often suffer from psychosocial sequelae like depression or poor self-image^4–6^. The major therapy options for synkinesis are facial training and a variety of physiotherapy approaches, medical treatment (mainly botulinum toxin treatment), and surgery^2^. Recently, we have shown that an intensified 10-day combined electromyography (EMG) and visual biofeedback training for patients with facial synkinesis leads to an improvement of facial function and reduced synkinesis^7^. The effects were stable over 6 months^7^. We decided to use this training as model for the present study.

We established high-resolution surface EMG (HR-sEMG) to describe the complex patterns of normal facial muscle activation and as a method to discriminate between different facial movements^8,9^. Using HR-sEMG, we could demonstrate that facial movement tasks elicit a complex pattern of muscle activity in patients with facial synkinesis. This pattern is partly functionally decoupled, involving both voluntary movements and involuntary muscle activation in areas adjacent to the target region and on the contralateral side^10^. We hypothesize that HR-sEMG cannot only be used as a diagnostic tool, but also as an objective method to evaluate the outcome of the combined visual and EMG biofeedback training. Therefore, the overall facial muscle activity, as well as the activity in each facial muscle at rest and during facial movement tasks, were measured in patients with facial synkinesis and hypertonicity due to postparalytic facial nerve syndrome, both before and at the end of the 10-day intensive training course. The method and results presented here can provide an objective method for the evaluation of facial muscle training. It might be even used for tailoring a personalized therapy targeting the individual facial synkinesis and hypertonicity patterns.

## Materials and methods

### Patients

The study included 36 patients (81% women; age range: 24–70 years) suffering from a postparalytic facial nerve syndrome with synkinesis after prior peripheral facial palsy without any other current or past neurological disease. The patients were presented recently in more detail^10^. No facial-palsy specific training was allowed during the waiting time before the training. No facial surgery or botulinum toxin injections were applied during the biofeedback training period. All patients gave written informed consent to participate in the study. The ethics committee of the Jena University Hospital approved the prospective clinical observational and longitudinal study (No. 2019–1539-BO). Informed written consent was also obtained to publish the information/image(s) in an online open-access publication or similar platforms. All methods were performed in accordance with the relevant guidelines and regulations.

### The combined electromyography and visual facial biofeedback training

**Fig. 1:**
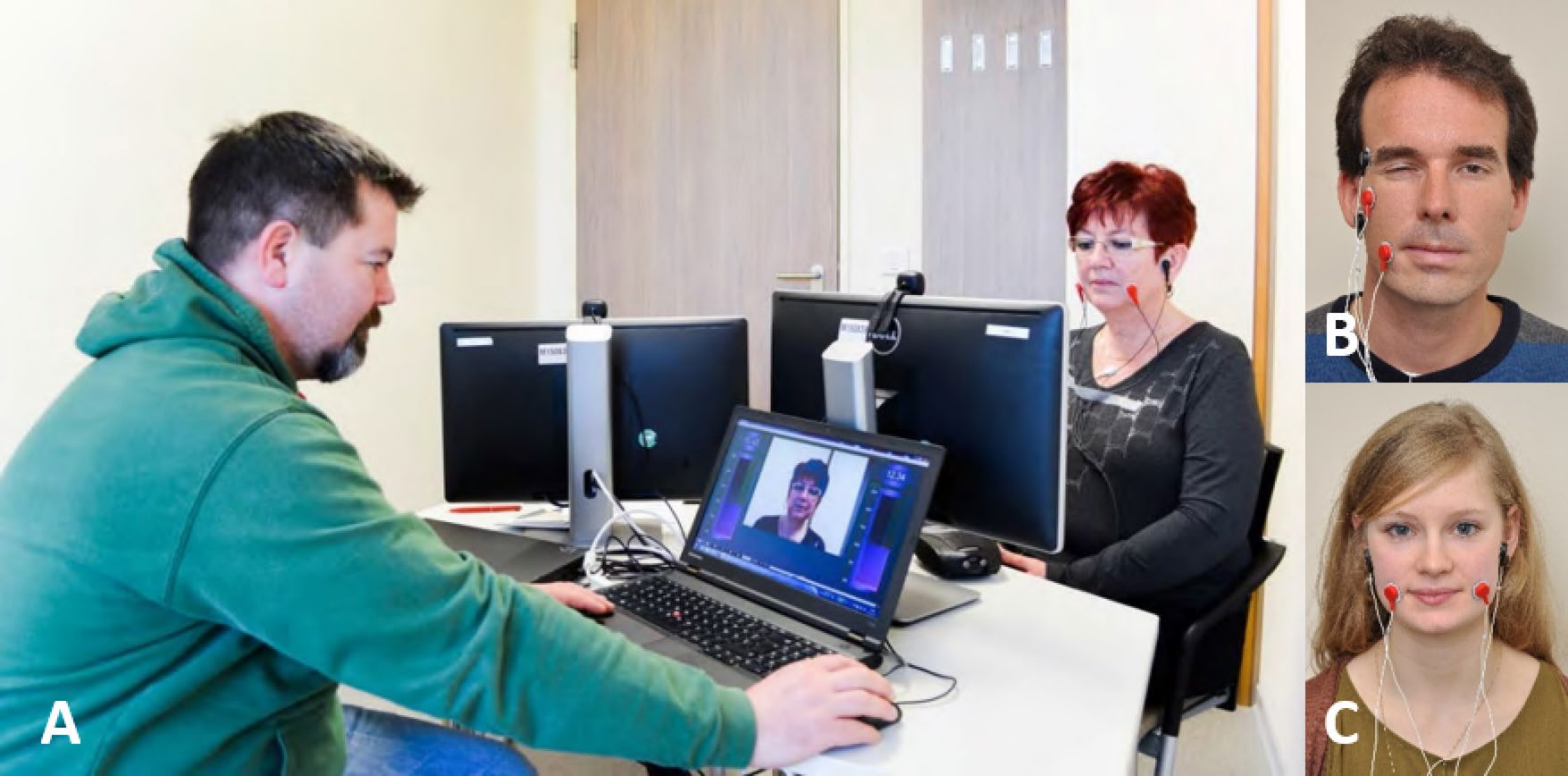
Setting of the combined visual and EMG biofeedback training. (**A**) The patient (right side) sits in front of a computer screen and the therapist (left side) has its own computer screen showing the same information as the screen of the patient. The patient sees herself/himself and the muscle activity of the recorded muscles is shown as bars on the left and right side of the screen. (**B**) Example of a unilateral setting on the synkinetic side: The task is to close the eye without activation of the mouth region. (**C**) Example of a bilateral setting: The task is to reach a symmetrical smile.

The training was described in detail elsewhere^7^. The training was carried out over a period of 10 days (two times for 5 days, the weekend in between without therapy). During every training day, the facial training with (EMG) biofeedback combined with elements of constraint induced movement therapy under the guidance of a therapist was carried out. The biofeedback training was performed using the Nexus 10 biofeedback system and Bio Trace software animations (Mind Media BV, Netherlands). Every patient trained continuously over 3 h per day to control a defined and isolated intended facial muscle movement (for instance, pursing the lips by activation of the orbicularis oris muscle) without moving other facial muscles (for instance, without unintended synkinetic activity of the ipsilateral orbicularis oculi muscle). Therefore, the two electrodes were placed on the ipsilateral orbicularis oris muscle and the orbicularis oculi muscle. The target muscles for the intended movement and for the unintended movement were monitored by bilateral surface EMG recording (Fig. 1). The respective muscle activity per movement was visualized with EMG feedback bars proportional to the muscle activity, therefore simultaneously enabling the patient to track the evoked muscle activity. This allowed a continuous demonstration of unconscious and unintended movements to the patient. The patient then attempted to lower the bar representing synkinetically activated muscle activity through conscious muscle relaxation, while simultaneously maintaining the bar representing the target muscle activity by performing the intended movement with controlled precision. Other tasks were focused on facial symmetry. For instance, the tasks was to smile symmetrically. Therefore, the two electrodes were placed on both zygomatic major muscles (Example in Supplementary Video S1). The therapist sat opposite the patient, monitoring both the patient and a video feed that displayed the patient’s image alongside the EMG feedback bars, identical to what the patient could see. This enabled the therapist to provide feedback on both progress and areas needing improvement, and to promptly adjust the movement exercises as necessary. The primary aim for patients was to develop new movement patterns with reduced synkinesis, allowing them to control their muscles independently and thereby balance their activity. Patients were requested to complement their supervised sessions each afternoon by two hours of individual training using a hand mirror^11^.

### High-resolution facial surface electromyography (HR-sEMG) registration

**Fig. 2:**
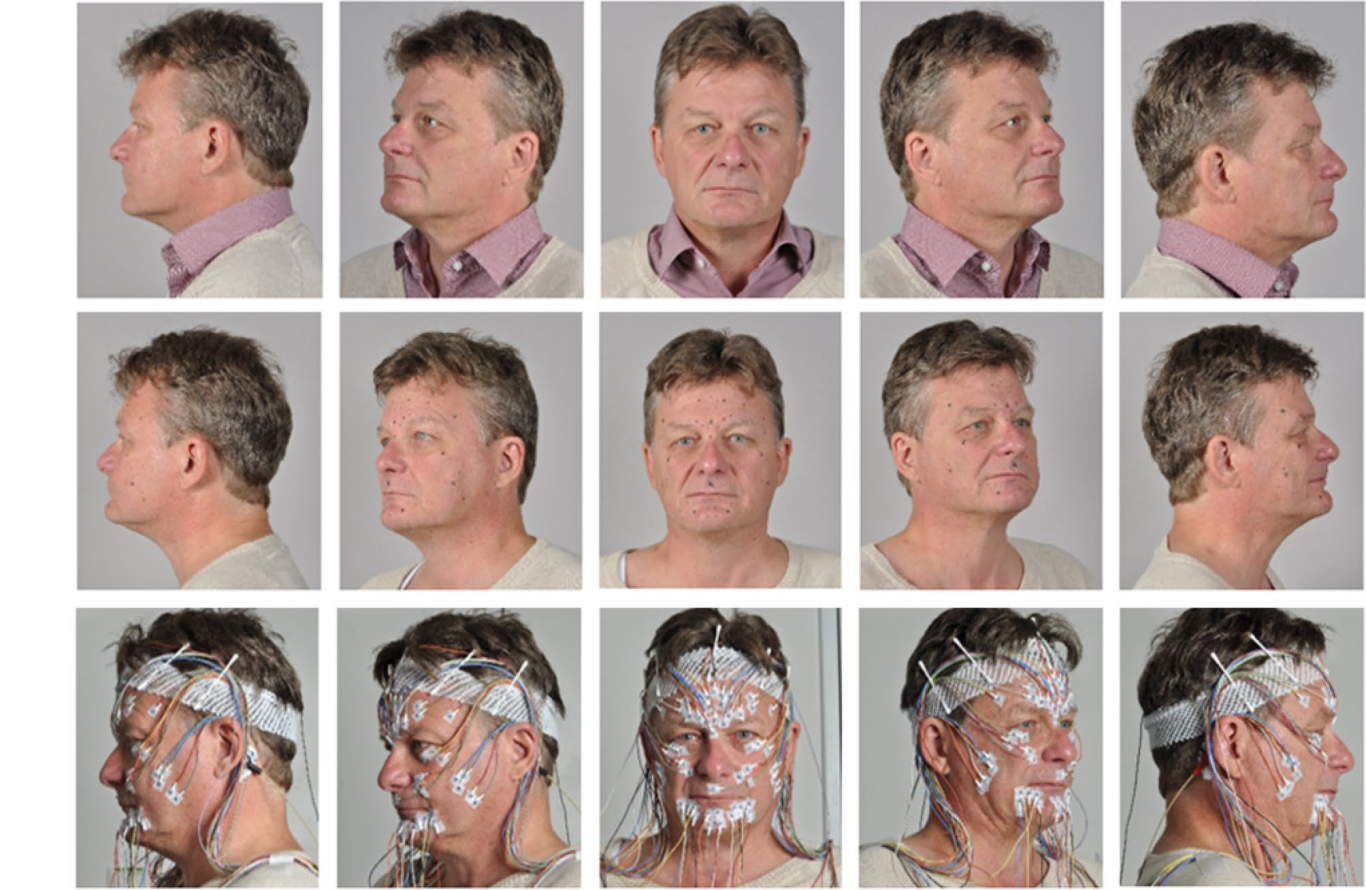
Standardized marking and electrode placement for the Fridlund and the Kuramoto scheme at the same time. Upper row: face at rest without any preparation. Middle row: The EMG electrode positions are marked for both the Fridlund and the Kuramoto scheme wit prefabricated stencils. Lower row: Complete set-up of surface EMG electrodes for both schemes.

HR-sEMG was performed synchronously and symmetrically from both sides of the face at the day before starting the training (T0) and on the penultimate day of training (T9) during display and imitated defined mimic expressions^8,9^. Monopolar surface electrodes were used (reusable Ag–AgCl discs, 6 mm diameter, DESS052606, GVB-geliMED, Bad Segeberg Germany). The reference electrodes were positioned at both mastoid processes and connected (disposable Ag–AgCl electrodes: H93 SG, Covidien, Neustadt, Germany). Two arrangements of electrodes described by Fridlund and Cacioppo^12^ and by Kuramoto et al.^13^ were used in parallel. The electrode positions of both EMG schemes are shown in Fig. 2. Overall, 58 electrodes were placed on the face (including one ground and the two connected reference electrodes). The HR-sEMG recording was performed with a multi-channel EMG system (gain: 100, frequency range 10–1,861 Hz; sampling rate 4,096/s; resolution: 5.96 nV/bit; DeMeTec, Langgöns, Germany).

### EMG data signal processing

The raw EMG data was processed using the ATISApro software (GJB Datentechnik GmbH, Langewiesen, Germany). As each of the two analog-to-digital converters generated its own measurement file, these files were first merged to create a single file containing all electrode derivations for each facial expression measurement. This was followed by a series of data processing steps called ‘chain’. The first step was an offset correction to eliminate possible deviations from the zero line. For the Fridlund scheme, subsequently bipolar channels were calculated by subtracting the signals from the respective pairs of the monopolarly measured electrodes^12^. Data for the Kuramoto scheme were monopolarly analyzed. A 50 Hz notch filter was then used to avoid interference due to mains noise. Finally, a bandpass filter (FIR filter) was used to eliminate unusable signal frequencies below 10 Hz and above 500 Hz. Each individual measurement file was now ready for the calculation of the mean amplitude values for each individual movement. The movement repetitions per facial expression (see below) were marked manually and output as root mean square (RMS) values. The RMS values were calculated consecutively at intervals of 0.125 s. The sampling rate of 4096/s corresponded to 512 measured sEMG values that were used to calculate one interval. All RMS mean values of the marked areas were then averaged individually for each evaluation channel and compiled in a data collection using Excel software (version 2016, Microsoft, Redmond, Washington, USA).

### Mimic exercises during the HR-sEMG registration

Once the electrodes had been applied, the patient was placed 80 cm in front of the examination screen and asked to follow the instructions in the instruction video with self-tutorial^14^. This video lasted approximately 15 min. A total of 11 different facial expressions were demonstrated. Each facial movement was repeated three times by the patients when prompted. The following movement exercises were performed: Face at rest (R; no movement), wrinkling of the forehead (WF), closing the eyes normally (CEN), closing the eyes forcefully (CEF), wrinkling the nose (WN), smiling with closed mouth (CMS), smiling with open mouth (OMS), puckering the lips (lip pursing; LP), blowing-out the cheeks (BC), snarling (S), and depressing the lower lip (DLL).

### Statistics

Microvolt-scaled mean values ± 95% confidence intervals were calculated for the patient sample. The statistical analysis was performed using SPSS (IBM, Armonk, New York, USA). A linear mixed-effects model (LMM) was used with the main effects of time of examination, side of face, facial expression, electrode position and repetition of movement per facial expression, as well as their bilateral interactions. Systematic differences were calculated with a significance level of *p* < 0.05 including correction for multiple testing. F values were used to determine the quantity of the variance (spread) between the mean values of the groups to be compared (e.g. affected and contralateral half of the face) in relation to the variance of the values within these groups. A large difference between the mean values of the compared groups therefore resulted in a high F value and thus also a high statistical significance.

## Results

### Comparison of the overall facial muscle activation before and at the end of the biofeedback training

**Fig. 3:**
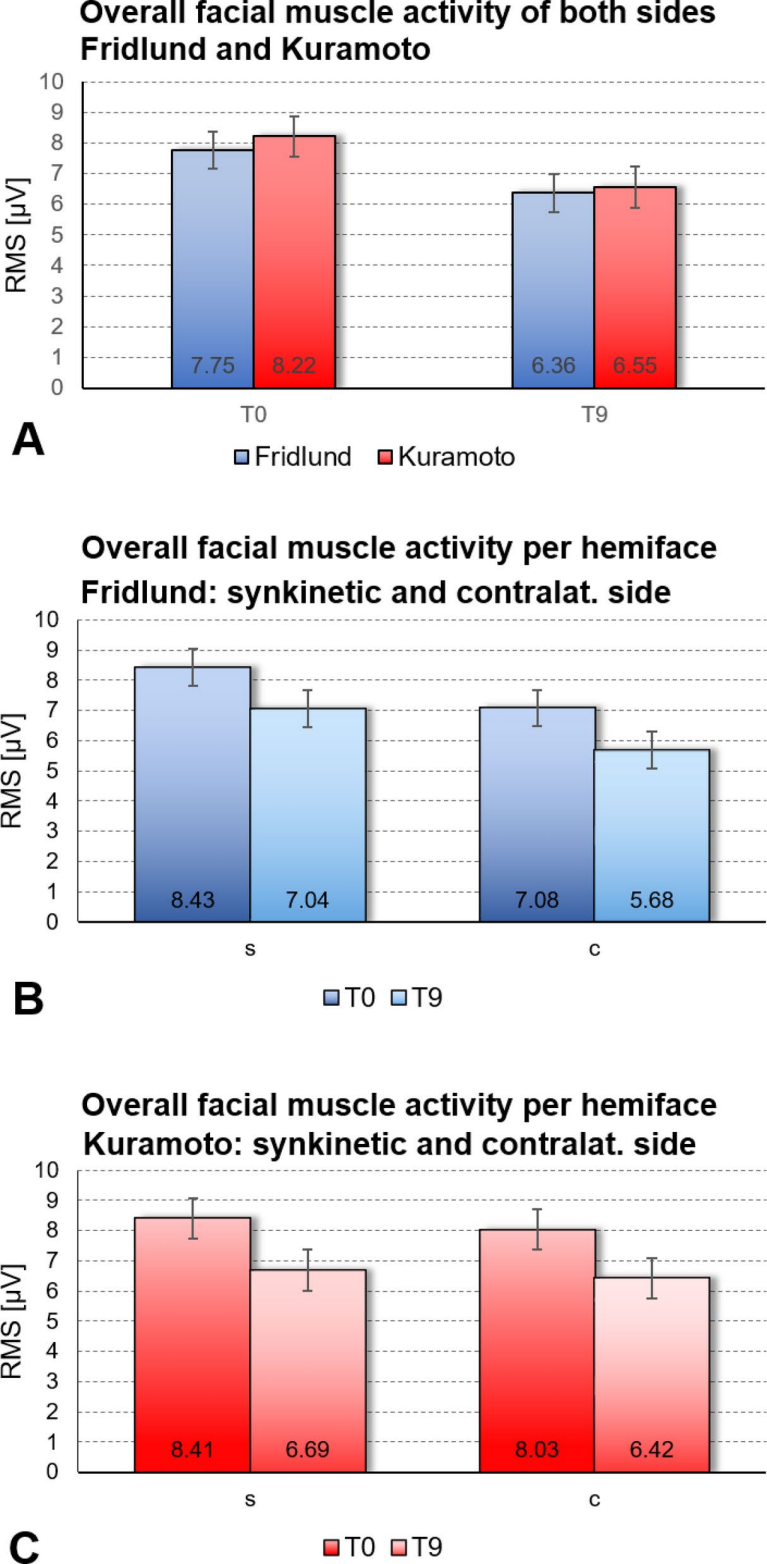
Average facial muscle activity (root mean square (RMS) in µV) of all recorded facial electrode positions (Mean ± 95% confidence interval) using the Fridlund EMG recording scheme (blue) and using the Kuramoto EMG recording scheme (red). (**A**) Overall activity at baseline (T0) and at the end of the training (T9). Both facial sides are averaged. (**B**) Overall activity using the Fridlund scheme separately fo the synkinetic (s) side and the contralateral (c) side at T0 and T9. **C**: Overall activity using the Kuramoto scheme separately fo the synkinetic (s) side and the contralateral (c) side at T0 and T9.

Figure 3A shows the averaged RMS values of all facial muscles (independent of side, Mimic exercise, and electrode positions) at T0 and T9 for the Fridlund and the Kuramoto electrode schemes. The overall muscle amplitude is a summatory marker for the influence of the biofeedback training on the relaxation of the entire facial musculature. In general, activity decreased significantly during the biofeedback training. (Fridlund scheme: F = 717.73; *p* < 0.001; Kuramoto scheme: F = 731.81; *p* < 0.001). This remained when both facial sides were evaluated separately: Using the Fridlund scheme data (Fig. 3B), the overall muscle activity from T0 to T9 decreased on the synkinetic side (F = 378.09; *p* < 0.001) and also on the contralateral side (F = 342.02; *p* < 0.001). The muscle activity was higher on the synkinetic side compared to the contralateral side at T0 (F = 325.57; *p* < 0.001) and also at T9 (F = 337.35; *p* < 0.001). Using the Kuramoto scheme similar results could be observed (Fig. 3C). The overall muscle activity decreased on the synkinetic side (F = 415.67; *p* < 0.001) and on the contralateral side (F = 322.52; *p* < 0.001). The muscle activity was again higher on the synkinetic side compared to the contralateral side at T0 (F = 18.63; *p* < 0.001) and also at T9 (F = 9.20; *p* = 0.002). The effect of the training on the decrease of the muscle activity was higher on the synkinetic side compared to the contralateral side by using the Kuramoto scheme.

### Overall facial muscle activation during the different mimic tasks

**Fig. 4:**
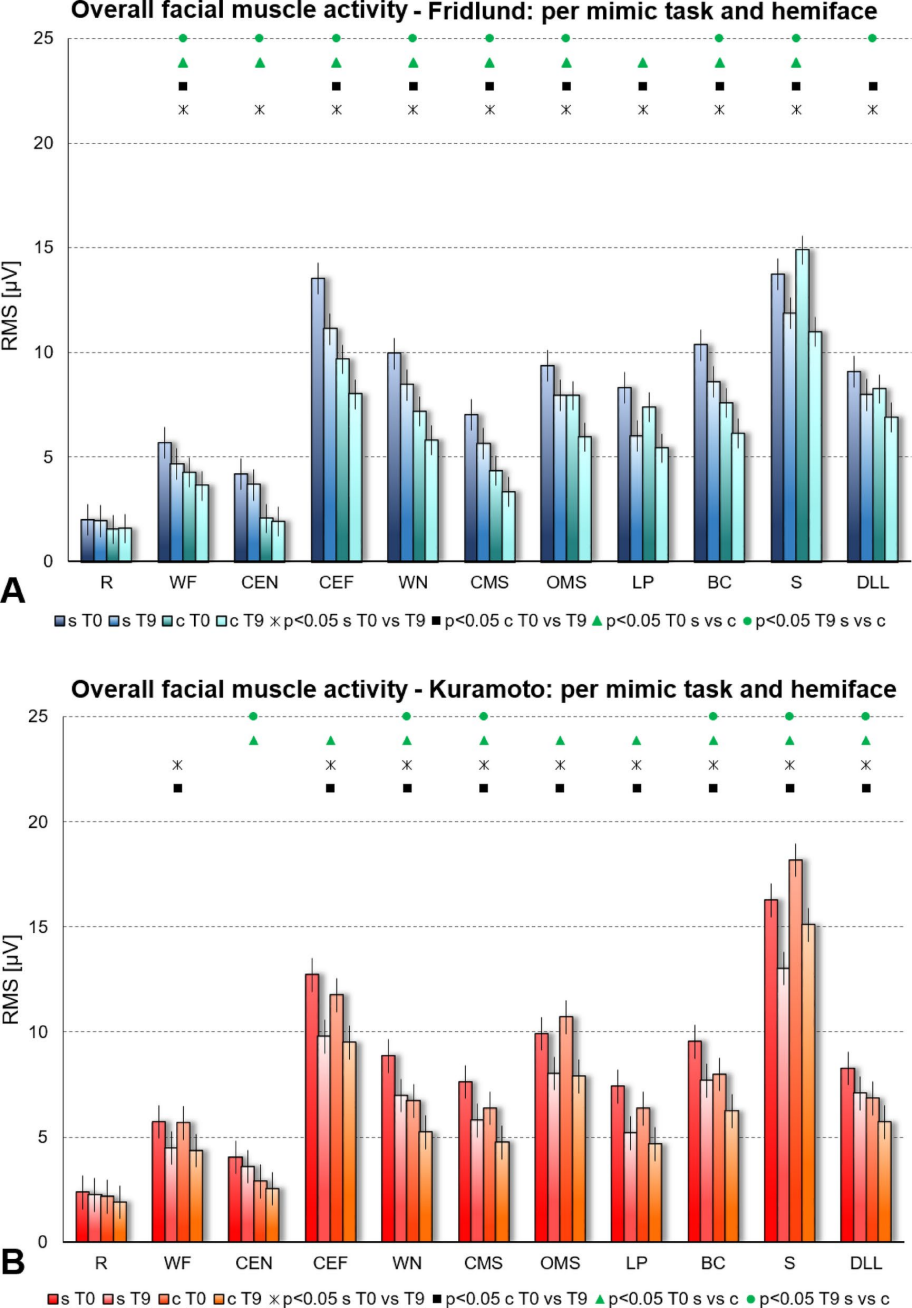
Average facial muscle activity (root mean square (RMS) in µV) of all recorded electrode positions (Mean ± 95% confidence interval) per facial exercise and hemiface. s = synkinetic side, c = contralateral side; T0 = baseline; T9 = at the end of training. The statistical comparisons are symbolized above the bars with different icons: Asterisk = synkinetic side: T0 versus T9; Box = contralateral side: T0 versus T9; Triangle = T0: synkinetic versus contralateral side; bullet = T9: synkinetic versus contralateral side. (**A**) Using the Fridlund EMG recording scheme (blue). (**B**) Using the Kuramoto EMG recording scheme (red). The facial movement tasks were: R = at rest; WF = Wrinkling of the forehead; CEN = Closing the eyes normally; CEF = Closing the eyes forcefully; WN = Wrinkling of the nose; CMS = Closed mouth smiling; OMS = Open mouth smiling; LP = Lip puckering; BC = Blowing-out the cheeks; S = Snarling; DLL = Depressing lower lip.

The results are visualized for the Fridlund scheme in Fig. 4A and for the Kuramoto scheme in Fig. 4B. Both facial sides (synkinetic and contralateral side) as well as the results at T0 and T9 were compared. Analyzing the data of the Fridlund scheme and of the Kuramoto scheme showed comparable results: Almost always, the observed muscle activity was higher at T0 than at T9 on both facial sides. The overall activity was higher in the lower than in the upper face region. For all tasks (except S), the observed muscle activity was highest on the synkinetic side at T0. The second highest activity was seen for the synkinetic side at T9 except for OMS, LP, S, and DLL: here second highest activity was seen on the contralateral side at T0. Comparing the different mimic tasks, the muscle activity was lowest at rest and highest during snarling. The differences between both sides and between T0 and T9 were significantly different nearly for all tasks (Details in Tables 1, 2). An exception was the activity at rest. Here, no differences between both facial sides and both time points were seen.

**Table 1:** Comparison of T0 versus T9 for the overall muscle activity for each functional facial task and per electrode positions separately for both facial sides.*

Fridlund	R	WF	CEN	CEF	WN	CMS	OMS	LP	BC	S	DLL
F-value, synkinetic side		22.80	5.57	128.02	48.87	41.87	43.49	115.31	68.72	76.53	27.33
*p*-value, synkinetic side		0.018	< 0.001	< 0.001	< 0.001	< 0.001	< 0.001	< 0.001	< 0.001	< 0.001	< 0.001
F-value, contralateral side		5.82		40.99	27.68	14.92	58.13	56.84	32.07	225.95	26.88
*p*-value, contralateral side		0.016		< 0.001	< 0.001	< 0.001	< 0.001	< 0.001	< 0.001	< 0.001	< 0.001

Kuramoto	R	WF	CEN	CEF	WN	CMS	OMS	LP	BC	S	DLL
F-value, synkinetic side		21.91		124.19	50.25	48.14	51.04	70.39	49.09	149.47	20.30
*p*-value, synkinetic side		< 0.001		< 0.001	< 0.001	< 0.001	< 0.001	< 0.001	< 0.001	< 0.001	< 0.001
F-value, contralateral side		19.93		60.30	26.38	30.44	92.08	34.17	36.31	112.10	14.86
*p*-value, contralateral side		< 0.001		< 0.001	< 0.001	< 0.001	< 0.001	< 0.001	< 0.001	< 0.001	< 0.001

Fridlund	DAO	OOr	Men	Mas	Zyg	LLS	OOc	LF	MF	CS	DS
F-value, synkinetic side	65.47	104.06	167.13	10.51	14.63	29.87	62.94	20.27	16.75	21.77	47.50
*p*-value, synkinetic side	< 0.001	< 0.001	< 0.001	0.001	< 0.001	< 0.001	< 0.001	< 0.001	< 0.001	< 0.001	< 0.001
F-value, contralateral side	48.00	164.41	190.42		12.65	27.08	41.77	4.26		5.38	11.90
*p*-value, contralateral side	< 0.001	< 0.001	< 0.001		< 0.001	< 0.001	< 0.001	0.039		0.02	0.001

Kuramoto	E9/10	E17/18	E15/16	E7/8	E5/6	E13/14	E3/4	E1/2			
F-value, synkinetic side	425.62	33.17	31.00	23.79	45.77	26.49	44.88	28.24			
*p*-value, synkinetic side	< 0.001	< 0.001	< 0.001	< 0.001	< 0.001	< 0.001	< 0.001	< 0.001			
F-value, contralateral side	234.36	45.14	30.56	24.31	31.40	23.51	23.28	17.32			
*p*-value, contralateral side	< 0.001	< 0.001	< 0.001	< 0.001	< 0.001	< 0.001	< 0.001	< 0.001			

*empty fields = no significant difference (p > 0.05), *R* at rest, *WF* Wrinkling of the forehead, *CEN* closing the eyes normally, *CEF* closing the eyes forcefully, *WN* wrinkling of the nose, *CMS* closed mouth smiling, *OMS* open mouth smiling, *LP* lip puckering, *BC* blowing-out the cheeks, *S* snarling, *DLL* depressing lower lip, *MF* frontal muscle. medial part, *LF* frontal muscle. lateral part, *CS* corrugator supercilii muscle, *DS* depressor supercilii muscle, *OOc* orbibularis oculi muscle, *Zyg* zygomatic muscle, *LLS* levator labii superioris muscle; LLS, *Mass* masseter muscle (not innervated by facial nerve. control muscle, *OOr* orbicularis oris muscle, *DAO* depressor anguli oris muscle, *Men* mentalis muscle.

**Table 2:** Comparison of synkinetic and contralateral side for the overall muscle activity for each functional facial task and per electrode positions separately for T0 and for T9.*

Fridlund	R	WF	CEN	CEF	WN	CMS	OMS	LP	BC	S	DLL
F-value, T0		17.47	48.21	187.61	86.71	81.80	17.38	5.20	87.69	34.85	
*p*-value, T0		< 0.001	< 0.001	< 0.001	< 0.001	< 0.001	< 0.001	0.023	< 0.001	< 0.001	
F-value, T9		12.39	44.45	163.33	114.69	83.41	60.29		96.47	7.27	12.94
*p*-value, T9		< 0.001	< 0.001	< 0.001	< 0.001	< 0.001	< 0.001		< 0.001	0.007	< 0.001

Kuramoto	R	WF	CEN	CEF	WN	CMS	OMS	LP	BC	S	DLL
F-value, T0			7.51	4.80	37.90	10.90	13.36	5.89	17.38	58.90	14.17
*p*-value, T0			0.006	0.028	< 0.001	0.002	< 0.001	0.015	< 0.001	< 0.001	< 0.001
F-value, T9			8.97		31.39	8.74			20.08	67.49	17.30
*p*-value, T9			0.003		< 0.001	0.003			< 0.001	< 0.001	< 0.001

Fridlund	DAO	OOr	Men	Mas	Zyg	LLS	OOc	LF	MF	CS	DS
F-value, T0	74.55		34.10				44.43	175.53	48.65	92.44	110.88
*p*-value, T0	< 0.001		< 0.001				< 0.001	< 0.001	< 0.001	< 0.001	< 0.001
F-value, T9	114.29	40.44	6.57		5.21	9.99	68.41	188.13	44.12	87.62	97.00
*p*-value, T9	< 0.001	< 0.001	0.01		0.022	0.002	< 0.001	< 0.001	< 0.001	< 0.001	< 0.001

Kuramoto	E9/10	E17/18	E15/16	E7/8	E5/6	E13/14	E3/4	E1/2			
F-value, T0	20.52				5.71		44.27	9.30			
*p*-value, T0	< 0.001				0.017		< 0.001	0.002			
F-value, T9	62.58		8.68		6.12	3.86	39.65	8.78			
*p*-value, T9	< 0.001		0.003		0.013	0.049	< 0.001	0.003			

*empty fields = no significant difference (p > 0.05), *R* at rest, *WF* Wrinkling of the forehead, *CEN* closing the eyes normally, *CEF* closing the eyes forcefully, *WN* wrinkling of the nose, *CMS* closed mouth smiling, *OMS* open mouth smiling, *LP* lip puckering, *BC* blowing-out the cheeks, *S* snarling, *DLL* depressing lower lip, *MF* frontal muscle. medial part, *LF* frontal muscle. lateral part, *CS* corrugator supercilii muscle, *DS* depressor supercilii muscle, *OOc* orbibularis oculi muscle, *Zyg* zygomatic muscle, *LLS* levator labii superioris muscle; LLS, *Mass* masseter muscle (not innervated by facial nerve. control muscle, *OOr* orbicularis oris muscle, *DAO* depressor anguli oris muscle, *Men* mentalis muscle.

### Overall facial muscle activation at different electrode positions

**Fig. 5:**
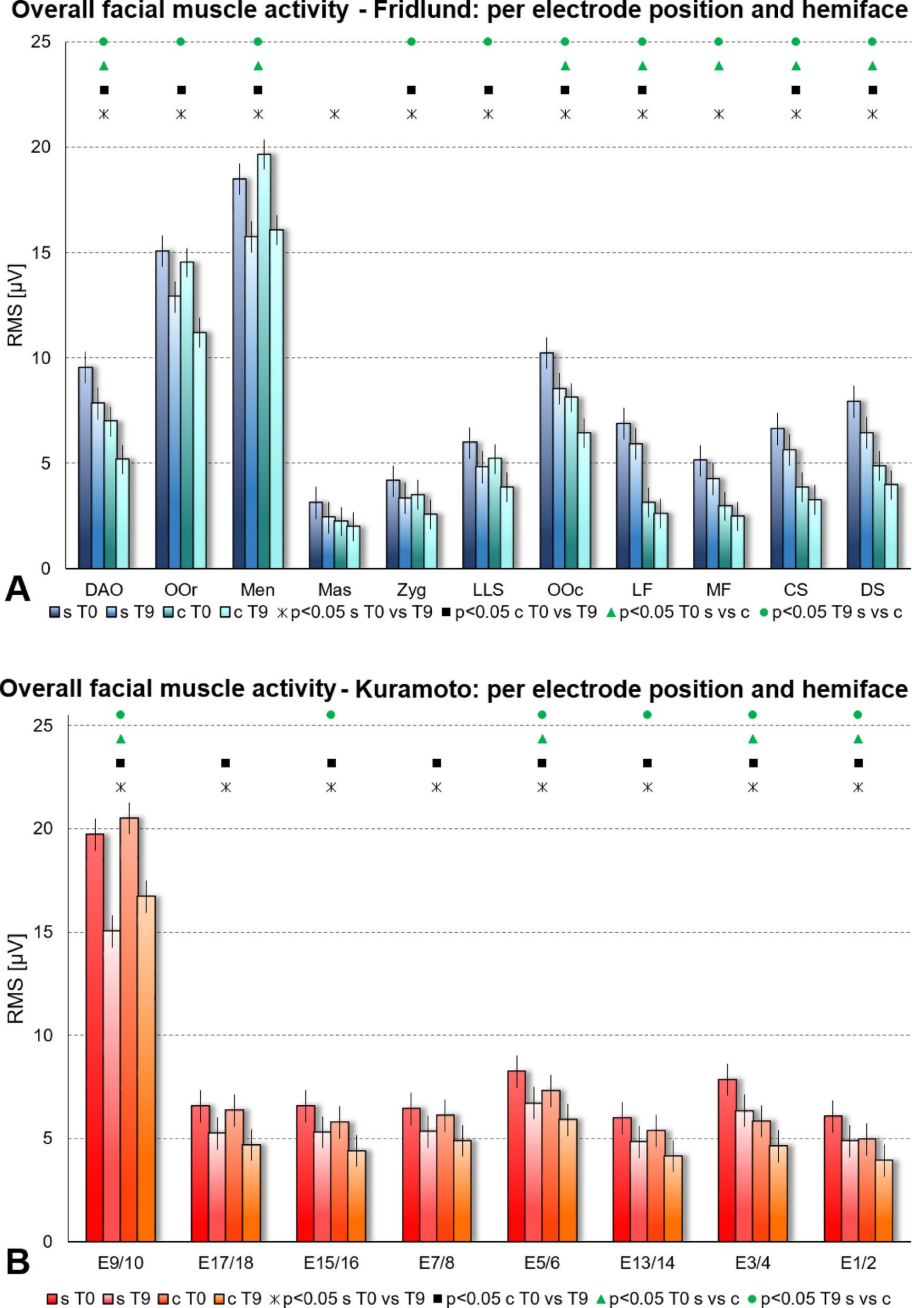
Average facial muscle activity (root mean square (RMS) in µV) of all recorded facial tasks (Mean ± 95% confidence interval) per electrode position and hemiface. s = synkinetic side, c = contralateral side; T0 = baseline; T9 = at the end of therapy. The four different columns in four different color intensities present the two time points and the facial sides s at T0, s at T9, c at T0, and c at T9. The statistical comparisons are symbolized above the bars with different icons: Asterisk = synkinetic side: T0 versus T9; Box = contralateral side: T0 versus T9; Triangle = T0: synkinetic versus contralateral side; bullet = T9: synkinetic versus contralateral side. (**A**) Using the Fridlund EMG recording scheme (blue). (**B**) Using the Kuramoto EMG recording scheme (red). The investigated facial muscles were: MF = frontal muscle. medial part; LF = frontal muscle. lateral part; Corr = corrugator supercilii muscle; DS = depressor supercilii muscle; OOc = orbibularis oculi muscle; Zyg = zygomatic muscle; LLS = levator labii superioris muscle; Mass = masseter muscle (not innervated by facial nerve. control muscle); OOr = orbicularis oris muscle; DAO = depressor anguli oris muscle; Men = mentalis muscle.

The results are visualized for the Fridlund scheme in Fig. 5A and for the Kuramoto scheme in Fig. 5B. Both facial sides (synkinetic and contralateral side) as well as the results at T0 and T9 were compared. Analyzing the data of the Fridlund scheme, the muscle activity was higher at T0 than at T9 on both facial sides expect for the Masseter muscle (only for the synkinetic side) The overall activity was higher in the lower than in the upper face. The muscle activity was highest for nearly all tasks on the synkinetic side at T0. The muscle activity was lowest in the masseter muscle (not innervated by the facial nerve) and highest in the mentalis muscle. The differences between both sides and between T0 and T9 were significantly different nearly for all mimic tasks (Details in Tables 1, 2).

Analyzing the data of the Kuramoto scheme, the muscle activity was also higher at T0 than at T9 for all electrode positions and independent of the side. The overall activity was not clearly higher in the lower than in the upper face. The muscle activity was highest for almost all tasks on the synkinetic side at T0. Electrode position E9/10 showed highest amplitudes, whereas all other electrode positions showed similar, but considerably lower amplitudes. Per mimic task, the differences on the synkinetic side between T0 and T9 were significantly different for all electrode positions, whereas a significant difference between both time points were seen only for a few electrode pairs on the contralateral side (Details in Tables 1, 2).

### Facial muscle activation at the different electrode positions per facial movement task

**Fig. 6:**
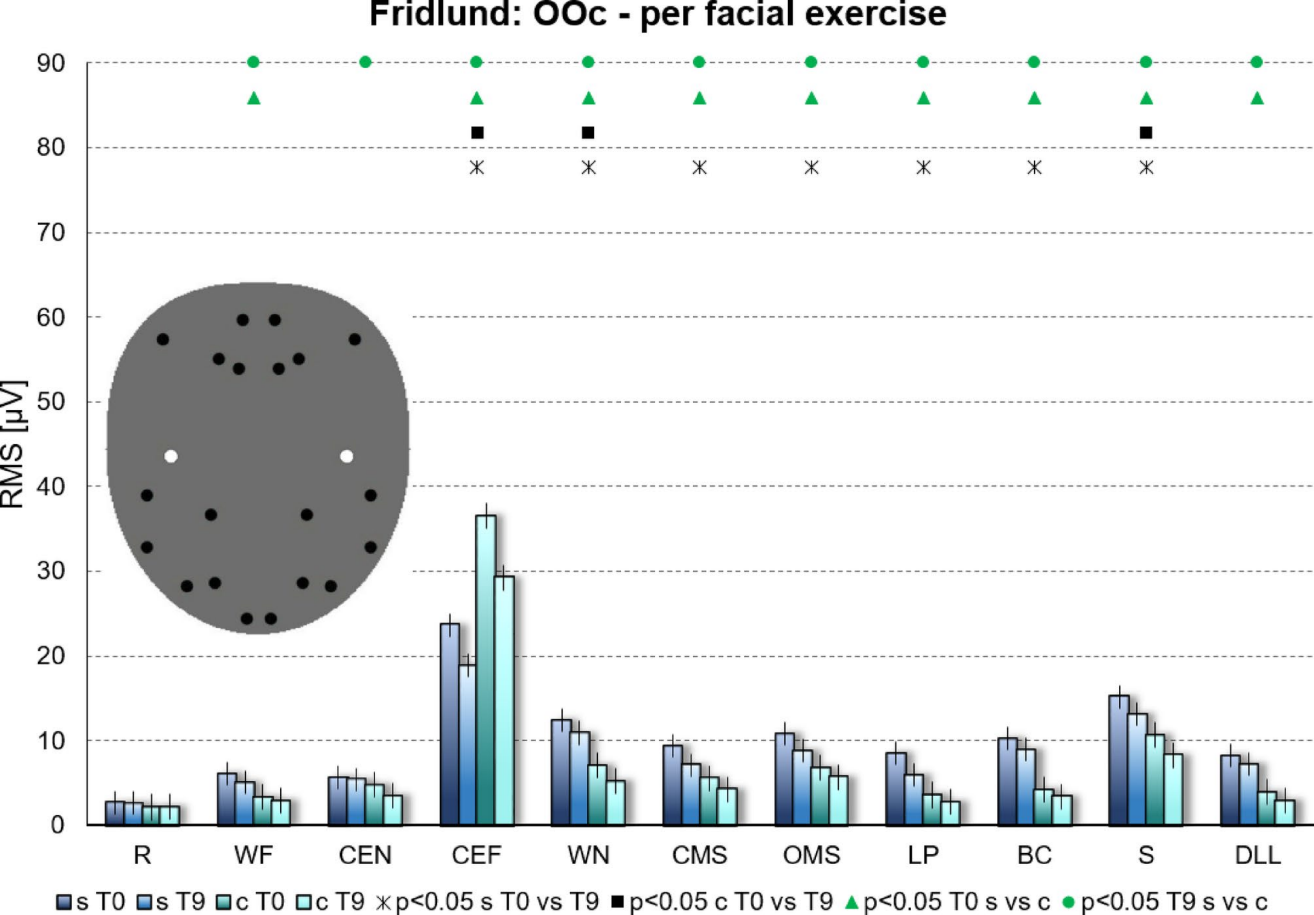
Facial muscle activation of the orbicularis oculi muscle (OOc) during each facial movement task for the Fridlund scheme. The facial muscle activation for all electrode positions are shown in Supplementary Figure S1. Maximal activation is seen during forced eye closing. Activation is also seen during many other facial movements, even in lower face tasks like snarling. This synkinetic activity decreased from T0 to T9. Average facial muscle activity (root mean square (RMS) in µV) in the OOc (Mean ± 95% confidence interval) per facial exercise and hemiface. s = synkinetic side, c = contralateral side; T0 = baseline; T9 = at the end of training. The statistical comparisons are symbolized above the bars with different icons: Asterisk = synkinetic side: T0 versus T9; Box = contralateral side: T0 versus T9; Triangle = T0: synkinetic versus contralateral side; bullet = T9: synkinetic versus contralateral side. (**A**) Using the Fridlund EMG recording scheme (blue). (**B**) Using the Kuramoto EMG recording scheme (red). The facial movement tasks were: R = at rest; WF = Wrinkling of the forehead; CEN = Closing the eyes normally; CEF = Closing the eyes forcefully; WN = Wrinkling of the nose; CMS = Closed mouth smiling; OMS = Open mouth smiling; LP = Lip puckering; BC = Blowing-out the cheeks; S = Snarling; DLL = Depressing lower lip.

In Supplementary Figure S1 (pages 3–8) the muscle activity for each recorded facial muscle is separately shown for each facial movement using the data of the Fridlund scheme. As one example, the muscle activity in the orbicularis oculi muscle (OOc) during all different exercises is shown in Fig. 6**.** This was important to understand the influence of the biofeedback training on the synkinetic activity. Significant differences (*p* < 0.05) between synkinetic versus contralateral side as well as for T0 versus T9 are indicated with symbols. More details of the statistical comparisons are shown in Supplementary Table S1 and Supplementary Table S2. The highest synkinetic activity in lower face muscle like the depressor anguli oris muscle (DAO), orbicularis oris muscle (OOr), and mentalis muscle (Men) were seen when the eyes were closed forcefully. The training significantly decreased this synkinetic activity. Vice versa, muscles in the upper face like the OOc, the frontalis muscle (LF and MF), the corrugator supercilii muscle (CS), and the depressor supercilii muscle (DS) showed the highest synkinetic activity during snarling (cf. Fig. 6). Again, this activy was reduced after the training. The muscles in the midface, i.e., the zygomatic muscle (Zyg) and the levator labii superioris muscle (LLS) showed also most synkinetic activity during forced eye closing, but much less than muscles of the upper face. Also here the synkinetic activity decreased significantly at the end of the training. The masseter muscle (Mas) showed a low activity during each facial exercise.

In Supplementary Figure S1 (pages 9–13) the muscle activity for each of Kuramoto’s electrode position is shown separately for each facial movement. Details on the statistical comparisons are shown in Supplementary Table S3 and Supplementary Table S4. The electrode positions of the Kuramoto scheme are not related to single facial muscles. There, a direct analysis of the synkinetic activity during the facial tasks was not feasible, but merely suggested it. Electrode pairs in the lower face like E9/10 or E17/18 showed activity during, for instance, forced eye closure. Vice versa, electrode pairs in the upper facie like E3/4 and E1/2 showed synkinetic activity when snarling. This synkinetic activity in all parts of the face were significantly reduced by the training.

## Discussion

As a safe and noninvasive tool for neuromuscular function evaluation, sEMG is widely used for neurological and muscle diagnostics and as part of EMG-biofeedback training in rehabilitation medicine^15^. Facial sEMG and especially HR-sEMG enables a detailed evaluation of the activity of motor units of the normal facial musculature or in patients with facial synkinesis^8,10^. This is worthwhile for facial diagnostics in patients with facial palsy or facial synkinesis or as real-time feedback element of a facial biofeedback training^7,16^. The more astonishing it is, that sEMG in form of HR-sEMG was used in the present study for the first time to objectively and quantitatively measure the effects of a facial rehabilitation training. As model we used a combined visual and sEMG biofeedback training on the muscle activity of patients with facial aberrant reinnervation^17^. This sequela of facial paralysis is characterized by muscle hypertonicity and synkinetic activation. Using HR-sEMG as a method for outcome measure, we could objectively show that a combined visual and EMG-based facial biofeedback training could successfully reduce the facial muscle activity on both facial sides within 9 days of training. The effect was stronger on the synkinetic side than on the contralateral side when using the data of the recordings with the Kuramoto scheme. Furthermore, HR-sEMG showed that synkinesis between the upper and lower face was reduced. The effects were shown with two different and independent EMG recording schemes. Finally, the effects seemed to be specific for the facial muscles, because the masseter muscle that was recorded as control muscle, did not show all these effects. This objective confirmation of the training effect is important as the positive effects of this special facial biofeedback training were shown so far only by subjective grading^7^ and by an assessment with patient reported outcome measures (PROMs)^18^.

Many other studies analyzing effects of training protocols for patients severely affected by facial paralysis and following conditions only use subjective methods like facial grading (for instance^19–21^) or PROMs (for instance^22–24^) as outcome measures. Due to the subjective evaluation by an observer or by the patient itself, the intra-subject and inter-subject reliability is limited^25^. In contrast, facial HR-sEMG has a high re-test reliability^9^. Facial nerve mapping with electrostimulation is also able to quantify facial synkinesis, but is less standardizable than HR-sEMG^26^. Another objective alternative is the use of machine-learning based image analysis. Such technology was used only in a few conservative facial therapy studies^27–30^. How much the published algorithms were also trained on patients with facial synkinesis is unknown for most studies. Recently, we demonstrated that automated blinking analysis could classify eye blinking in patients with synkinesis reliable^31^. Currently, a study is ongoing using automated blinking analysis also for outcome measure of facial therapy. It would also be worthwhile to combine this with the HR-sEMG set-up presented here.

The present single-arm study had limitations. A randomized controlled clinical trial with a sham-trained control group would clearly demonstrate a specific therapeutic effect. It can also not be excluded that practicing facial movements in front of a mirror without EMG feedback would yield comparable outcomes^30^. We were primarily interested in establishing HR-sEMG as an objective method of therapy evaluation rather than confirming the effectiveness of biofeedback therapy. Furthermore, the sample size was too small to analyze for gender and age effects on therapy success. All patients had severe facial synkinesis, but the interval between onset of the palsy and training was variable .The therapy effect was only measured at day 9 of the treatment. The consistency of the therapy effects measured by HR-sEMG are unknown. Ideally, the reduction in side differences between the two sides of the face as a result of the biofeedback training would persist over the long term. Based on facial grading measurements, the effects are stable at least for six months after therapy completion^7^. We need to check in future studies whether we still find the HR-sEMG effects after 6 months or longer. At least when constraint induced movement therapy elements are used for post-stroke rehabilitation, a long-term consistency of the effects for a year has been shown^32^. It has not yet been investigated whether a longer training period than 10 days has a stronger therapeutic effect.

The examination with HR-sEMG in the present form is time-consuming. The preparations for a measurement take about an hour. At the moment we are evaluating if the EMG recordings of the biofeedback training itself, so far only used for the real-time visualization during the training, also could be used for facial muscle hypertonicity evaluation^33^. Recently, we have shown that facial HR-sEMG can also be recorded with a smart skin technology consisting of wireless dry 16-electrodes applied as self-adhesive foils^34^. Therefore, it is planned to apply these innovative EMG foils also for the analysis of the effects of the presented biofeedback training.

## Conclusions

HR-sEMG is not just a diagnostic tool for the analysis of facial muscle function and dysfunction. It can also be used as an objective measure to analyze treatment effects in patients with facial synkinesis following unilateral facial paralysis. To show this for the first time in a prospective observational study on 36 patients with facial aberrant reinnervation syndrome, we used a standardized a sEMG-based biofeedback training as model. HR-sEMG confirmed ie that a combined visual and sEMG-based biofeedback training seems to reduce facial muscle hypertonicity and the degree of synkinetic facial muscle activity within 9 days of an intensified training. This has to be proven now in a randomized control trial with sham biofeedback training in a control arm.

## Electronic supplementary material

Below is the link to the electronic supplementary material.

Supplementary Material 1

Supplementary Material 2

Supplementary Material 3

Supplementary Material 4

## Data Availability

The original contributions presented in the study are included in the article/Supplementary material. Further inquiries can be directed to the corresponding author.
