# Supplementary material for: Synchronous surface electromyography as objective method to evaluate the outcome of a biofeedback training in patients with facial synkinesis
Source: Sci Rep. 2025 May 19;15:17335. doi: 10.1038/s41598-025-01278-7 (PMC12089512; doi:10.1038/s41598-025-01278-7)
Supplement: Supplementary file 2 — Supplementary Material 2 [file 41598_2025_1278_MOESM2_ESM.docx]

**Synchronous high-resolution surface electromyography as objective method to evaluate the outcome of a biofeedback training in patients with facial synkinesis**

Richard Schneider, Maren Schramm, Paul F. Funk, Gerd Fabian Volk, Christoph Anders, Orlando Guntinas-Lichius

**Supplementary Video S1**

The video gives an example of the combined visual and electromyography-based biofeedback training for patients with facial synkinesis used as model to establish electromyography also an objective measurement tool to evaluate the outcome of the training. The video shows an example of a symmetry training. The electrodes are place on both sides on the zygomatic muscle. The proband has the task to activate both muscles symmetrically. The proband sees herself on the video screen and also the muscle activity. The muscle activity is shown as bars on both sides of the screen. The task is now to smile and thereby activate both muscles in a way that the bars are at the same level. The therapist sits opposite to the probands and sees the same image. This helps the therapist to steer and motivate the proband as well as to correcting the motion sequence.
