# Supplementary material for: Synchronous surface electromyography as objective method to evaluate the outcome of a biofeedback training in patients with facial synkinesis
Source: Sci Rep. 2025 May 19;15:17335. doi: 10.1038/s41598-025-01278-7 (PMC12089512; doi:10.1038/s41598-025-01278-7)
Supplement: Supplementary file 3 — Supplementary Material 3 [file 41598_2025_1278_MOESM3_ESM.pdf]

Supplement Figures S1

On the next sides, first the averaged results for each exercise are shown for each facial muscle using the Fridlund EMG recording scheme. Thereafter the results for the Kuramoto scheme for each electrode pair positions are presented. Kuramoto midline electrodes not evaluated.
The recorded muscle or the recorded electrode positions, respectively, are indicated on the schematic drawing of the face in each figure.
The facial muscle activation is presented in root mean square (RMS) in µV as mean±95% confidence interval. Above each graph, the results of the statistical comparisons corrected for multiple testing are shown. Statistical differences are marked with different symbols.

Abbreviations:

Investigated facial muscles: MF = frontal muscle, medial part; ML = frontal muscle, lateral part; Corr = corrugator supercilii muscle; DS = depressor supercilii muscle; OOc = orbicularis oculi muscle; Zyg = zygomatic muscle; LLS = levator labii superioris muscle; LLS; Mass = masseter muscle (not innervated by facial nerve, control muscle); OOr = orbicularis oris muscle; DAO = depressor anguli oris muscle; Ment = mentalis muscle.

The facial movement tasks: R = at rest; WF = Wrinkling of the forehead; CEF = Closing the eyes normally; CEF = Closing the eyes forcefully; WN = Wrinkling of the nose; CMS = Closed mouth smiling; OMS = Open mouth smiling; LP = Lip puckering; BC = Blowing-out the cheeks; S = Snarling; DLL = Depressing lower lip.

Facial sides: s = synkinetic side; c = contralateral side

Electrode schemes
FRIDLUND electrodes abbreviated with related muscle names

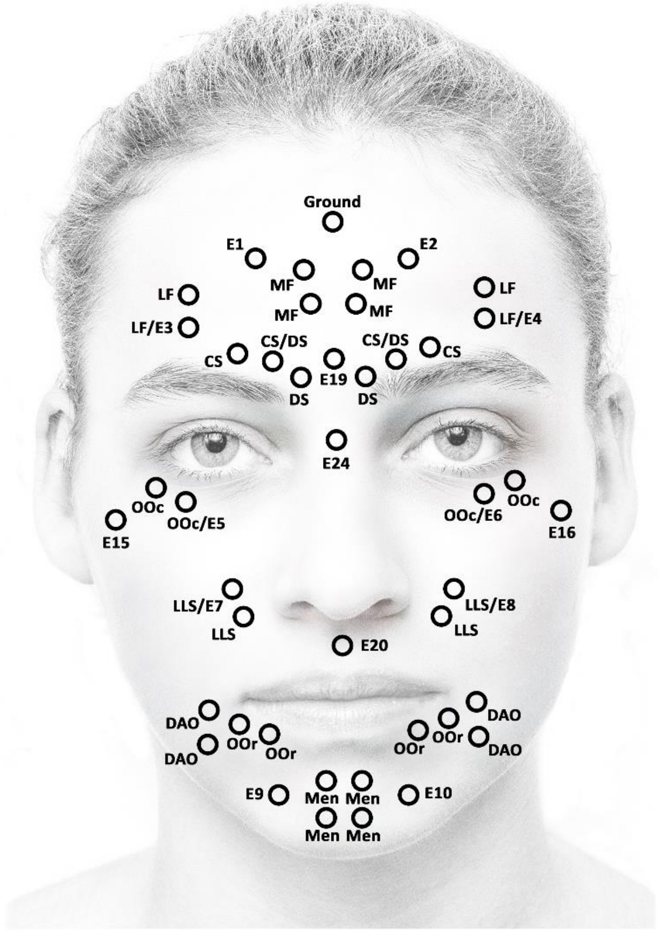

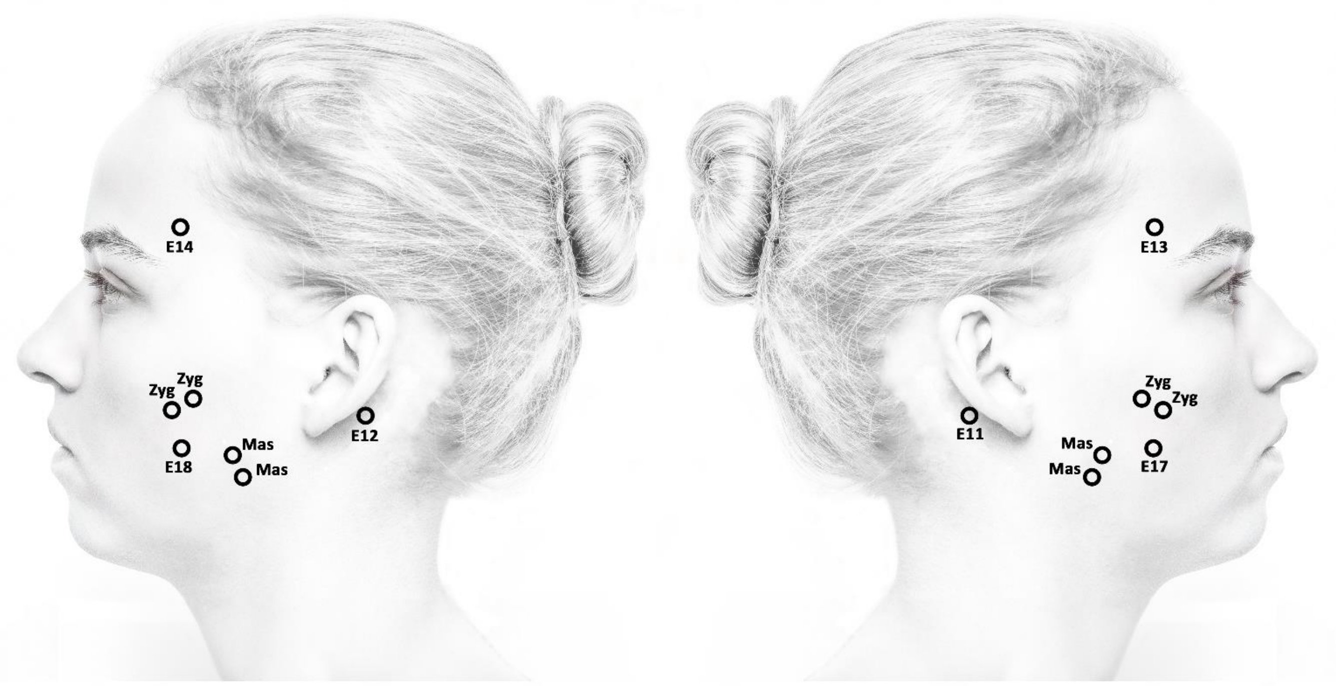

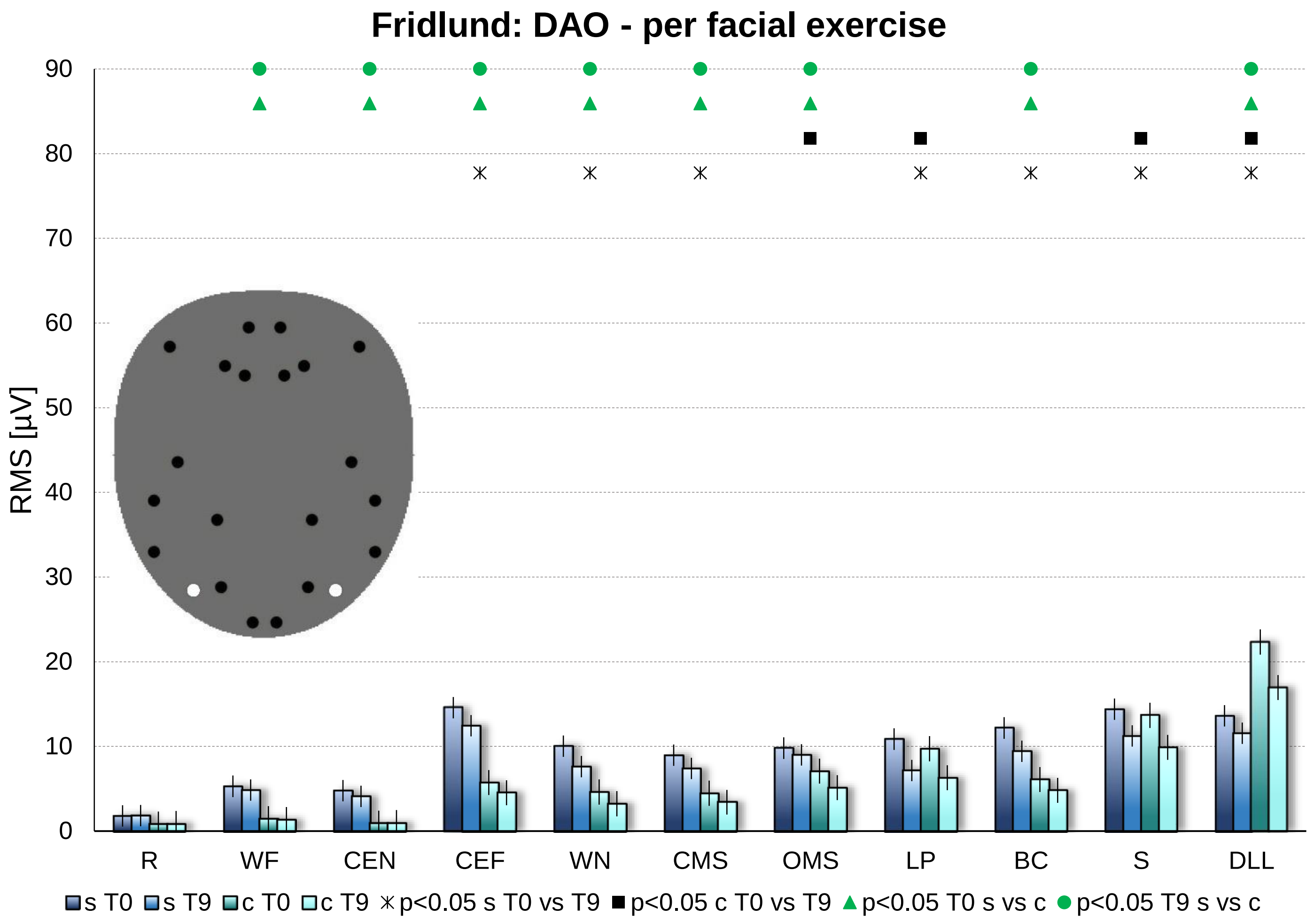

Fridlund: DAO - per facial exercise
RMS [µV]
90
80
70
60
50
40
30
20
10
0
R
WF
CEN
CEF
WN
CMS
OMS
LP
BC
S
DLL
s T0
s T9
c T0
c T9
p<0.05 s T0 vs T9
p<0.05 c T0 vs T9
p<0.05 T0 s vs c
p<0.05 T9 s vs c

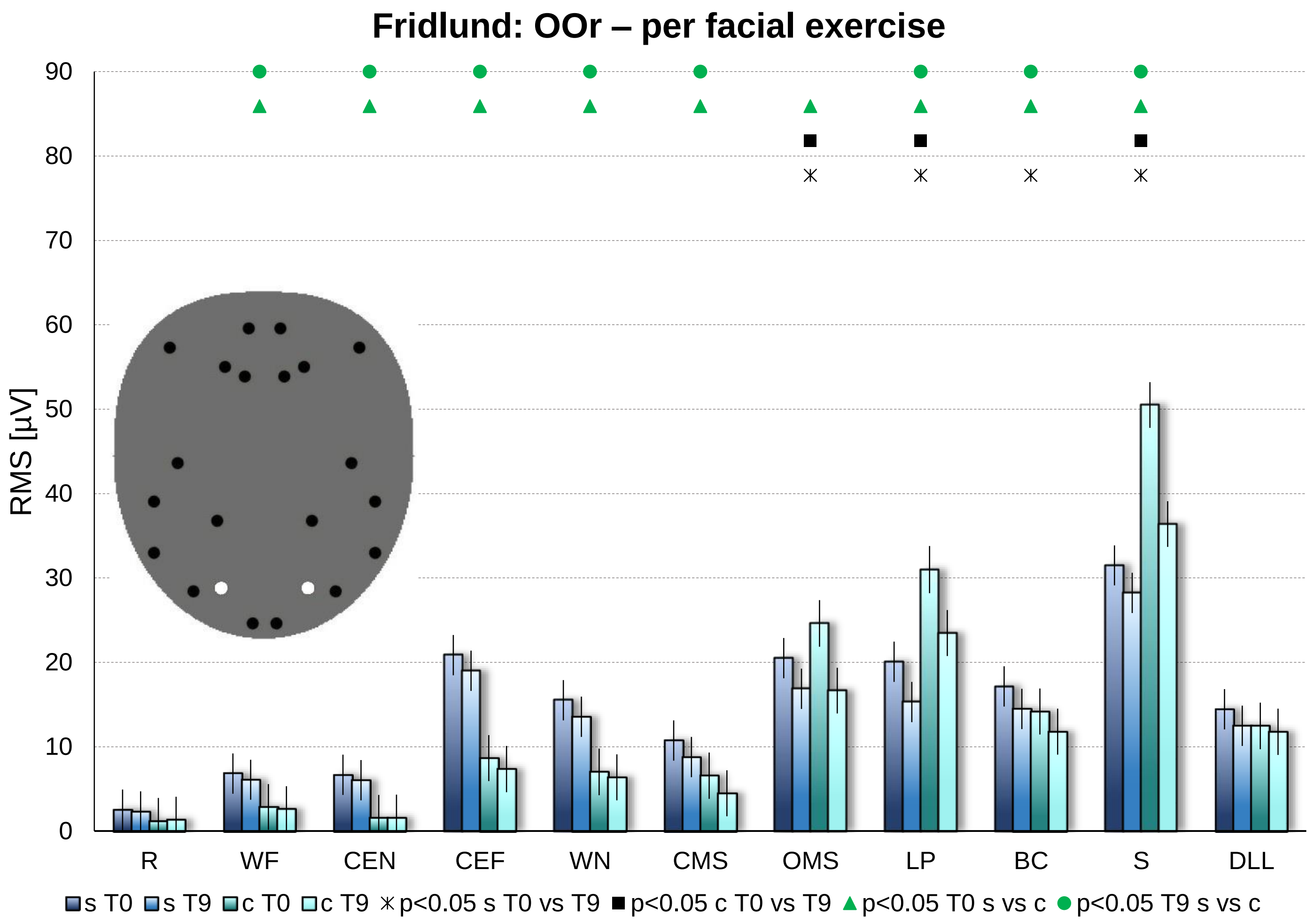

Fridlund: OOr – per facial exercise
RMS [µV]
90
80
70
60
50
40
30
20
10
0
R
WF
CEN
CEF
WN
CMS
OMS
LP
BC
S
DLL
s T0
s T9
c T0
c T9
p<0.05 s T0 vs T9
p<0.05 c T0 vs T9
p<0.05 T0 s vs c
p<0.05 T9 s vs c

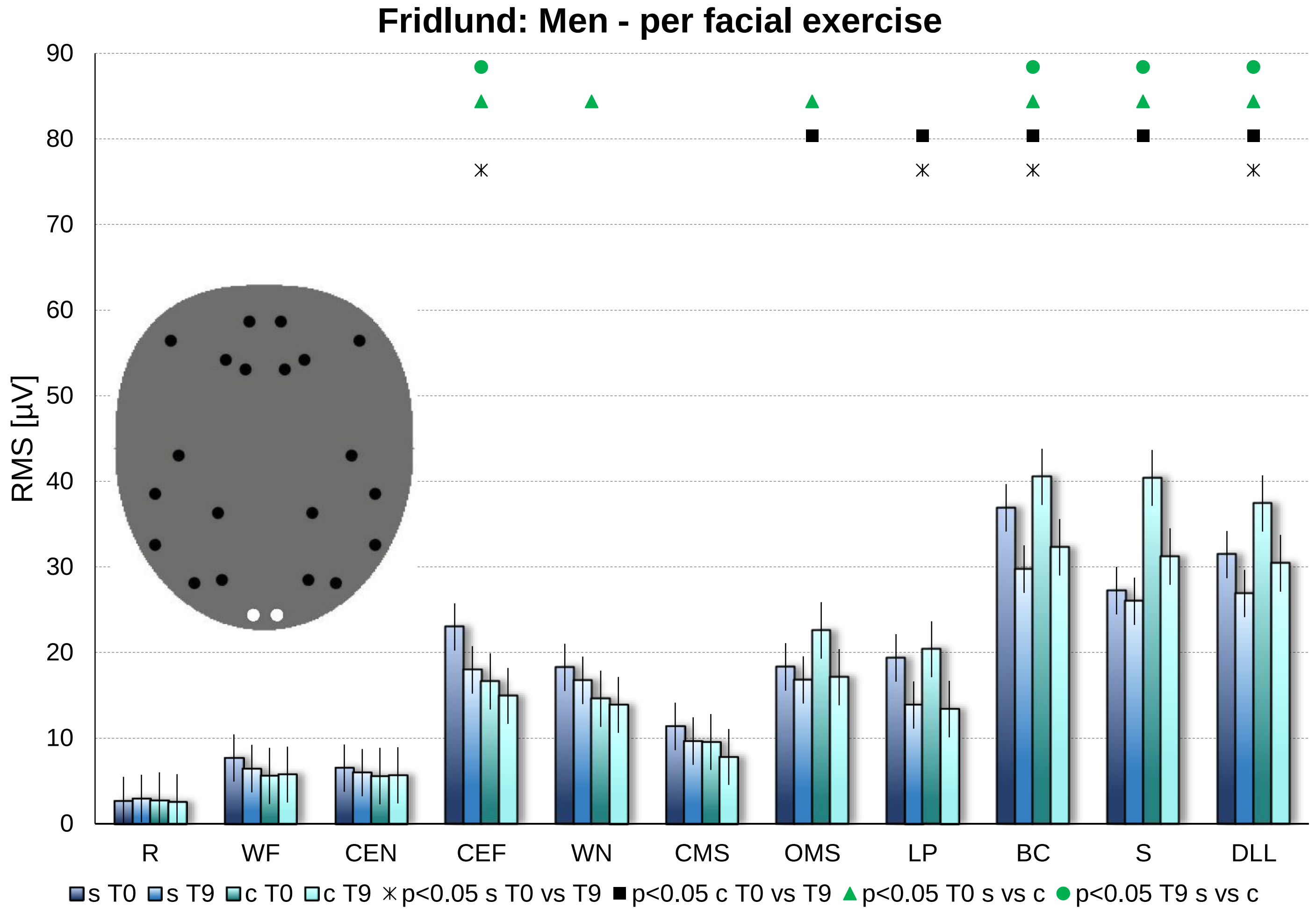
Fridlund: Men - per facial exercise
RMS [µV]
0
10
20
30
40
50
60
70
80
90
R
WF
CEN
CEF
WN
CMS
OMS
LP
BC
S
DLL
s T0
s T9
c T0
c T9
p<0.05 s T0 vs T9
p<0.05 c T0 vs T9
p<0.05 T0 s vs c
p<0.05 T9 s vs c

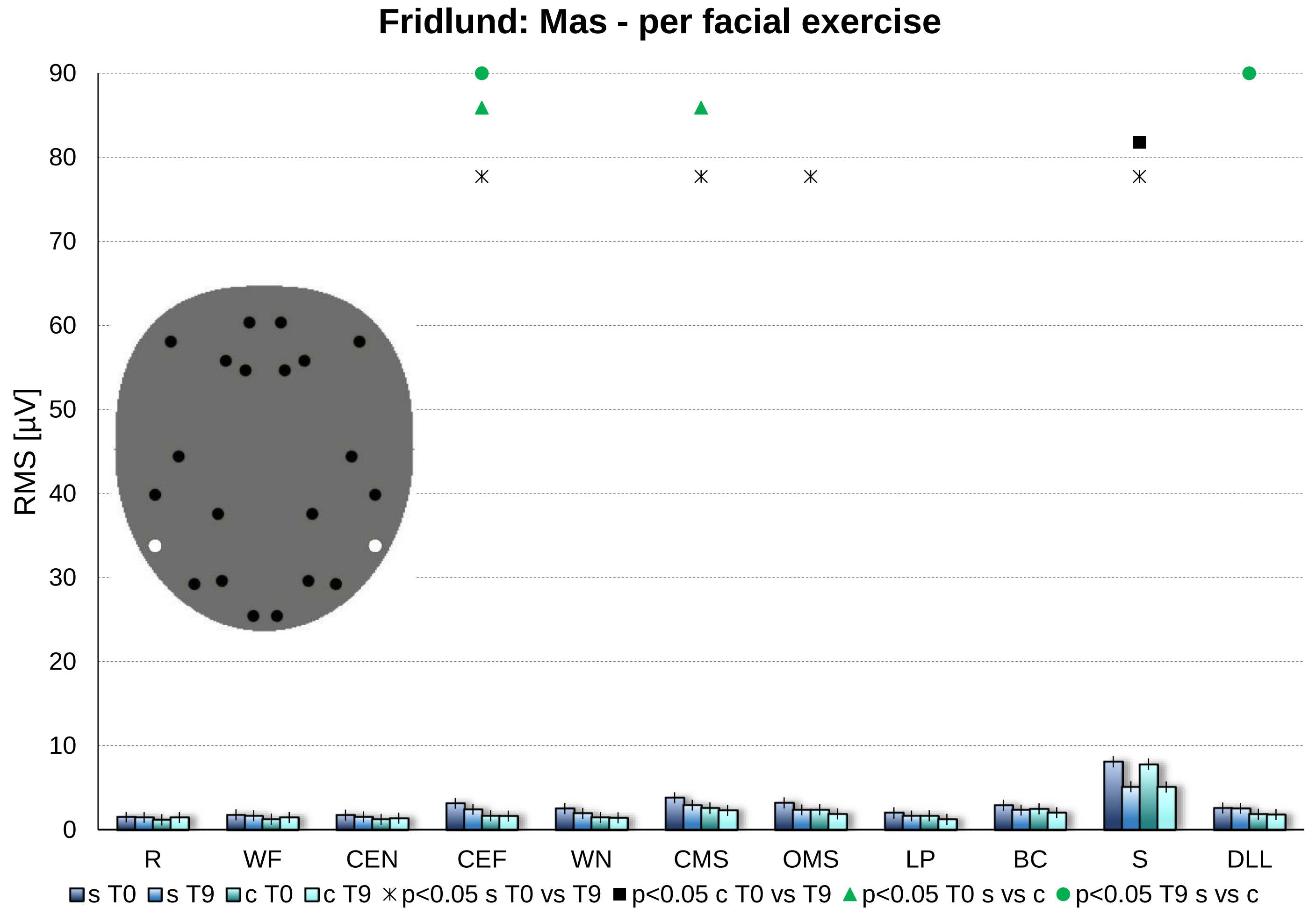
Fridlund: Mas - per facial exercise
RMS [µV]
0
10
20
30
40
50
60
70
80
90
R
WF
CEN
CEF
WN
CMS
OMS
LP
BC
S
DLL
s T0
s T9
c T0
c T9
p<0.05 s T0 vs T9
p<0.05 c T0 vs T9
p<0.05 T0 s vs c
p<0.05 T9 s vs c

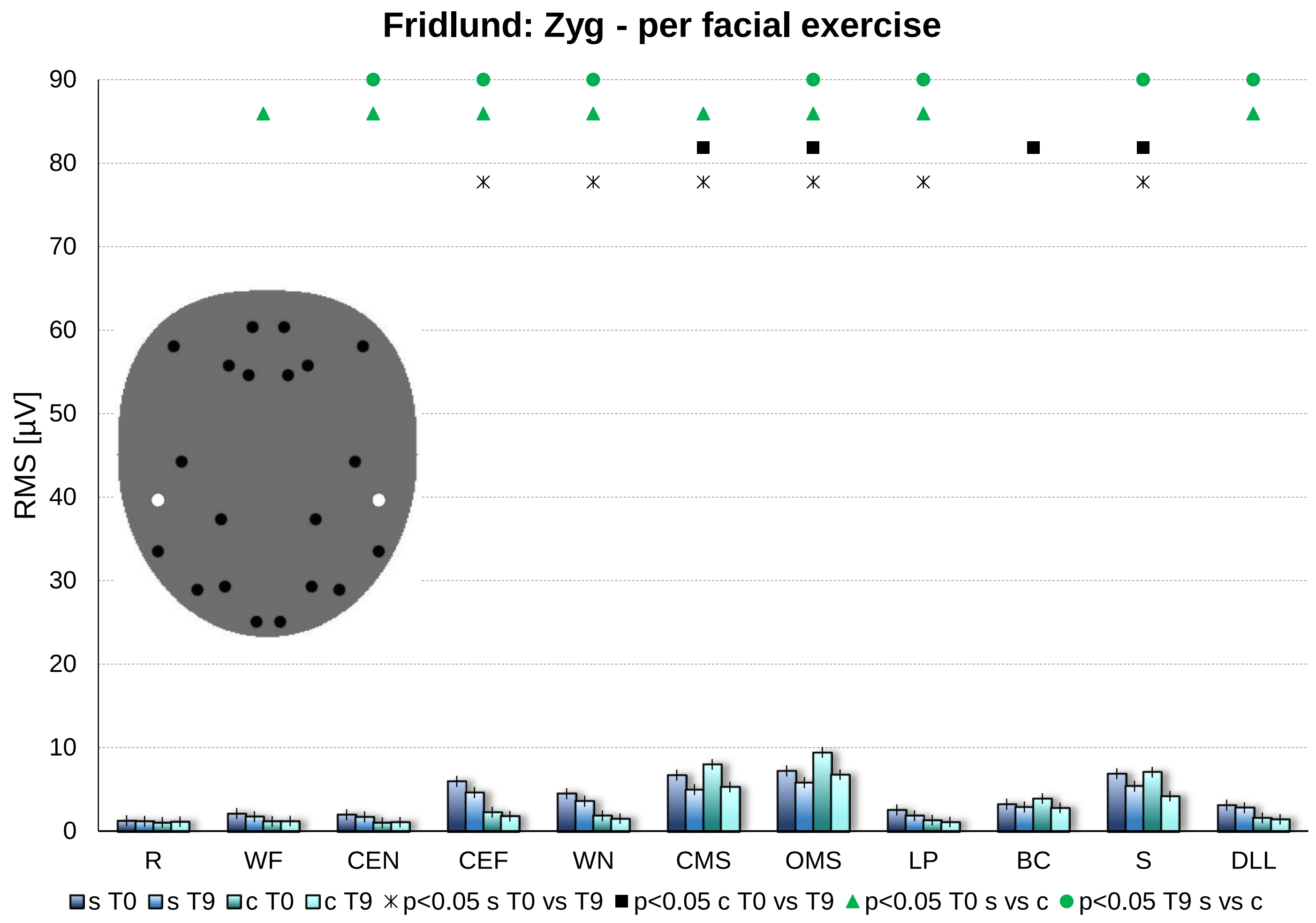

Fridlund: Zyg - per facial exercise
RMS [µV]
0
10
20
30
40
50
60
70
80
90
R
WF
CEN
CEF
WN
CMS
OMS
LP
BC
S
DLL
s T0
s T9
c T0
c T9
p<0.05 s T0 vs T9
p<0.05 c T0 vs T9
p<0.05 T0 s vs c
p<0.05 T9 s vs c

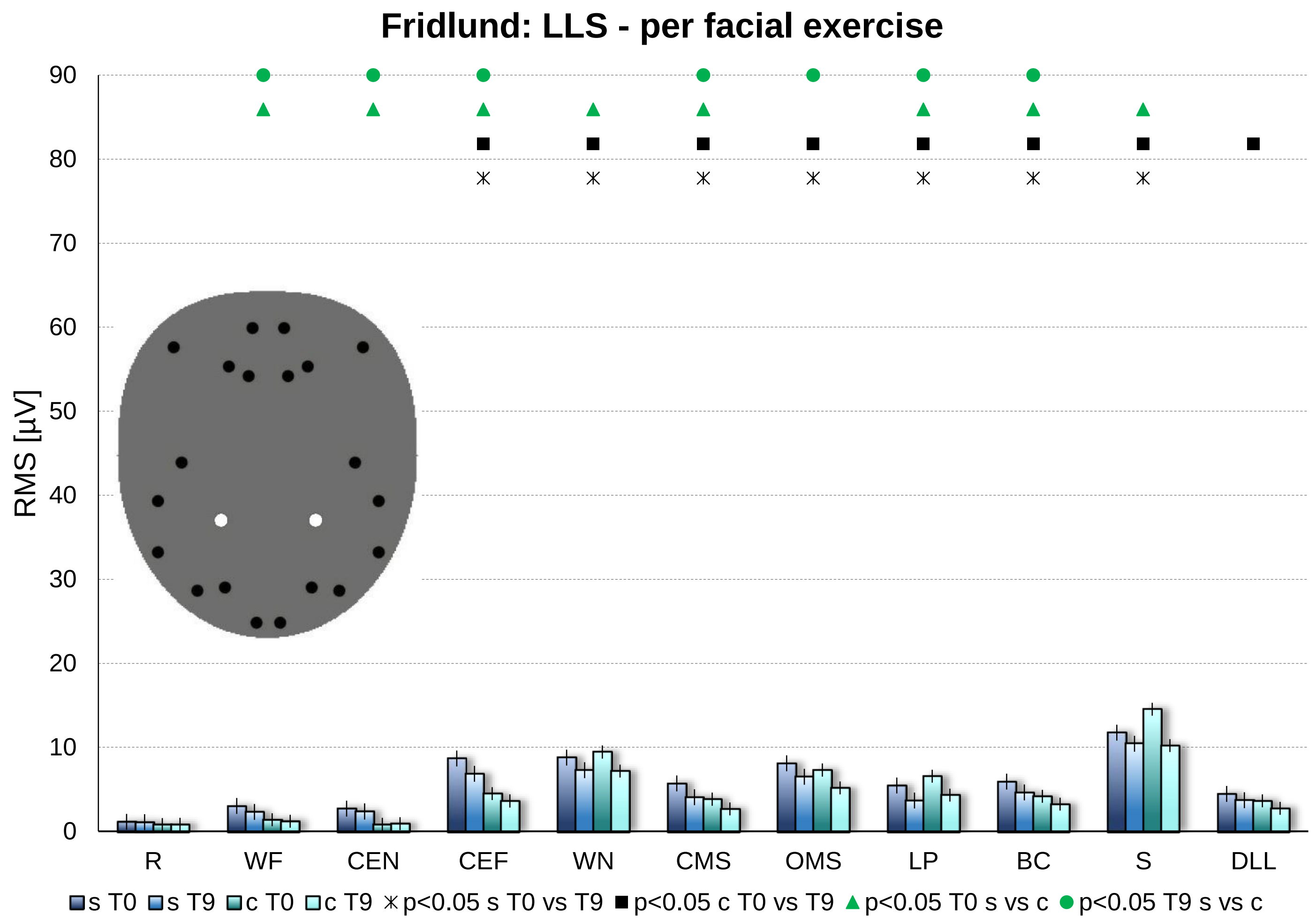

Fridlund: LLS - per facial exercise
RMS [µV]
0
10
20
30
40
50
60
70
80
90
R
WF
CEN
CEF
WN
CMS
OMS
LP
BC
S
DLL
s T0
s T9
c T0
c T9
p<0.05 s T0 vs T9
p<0.05 c T0 vs T9
p<0.05 T0 s vs c
p<0.05 T9 s vs c

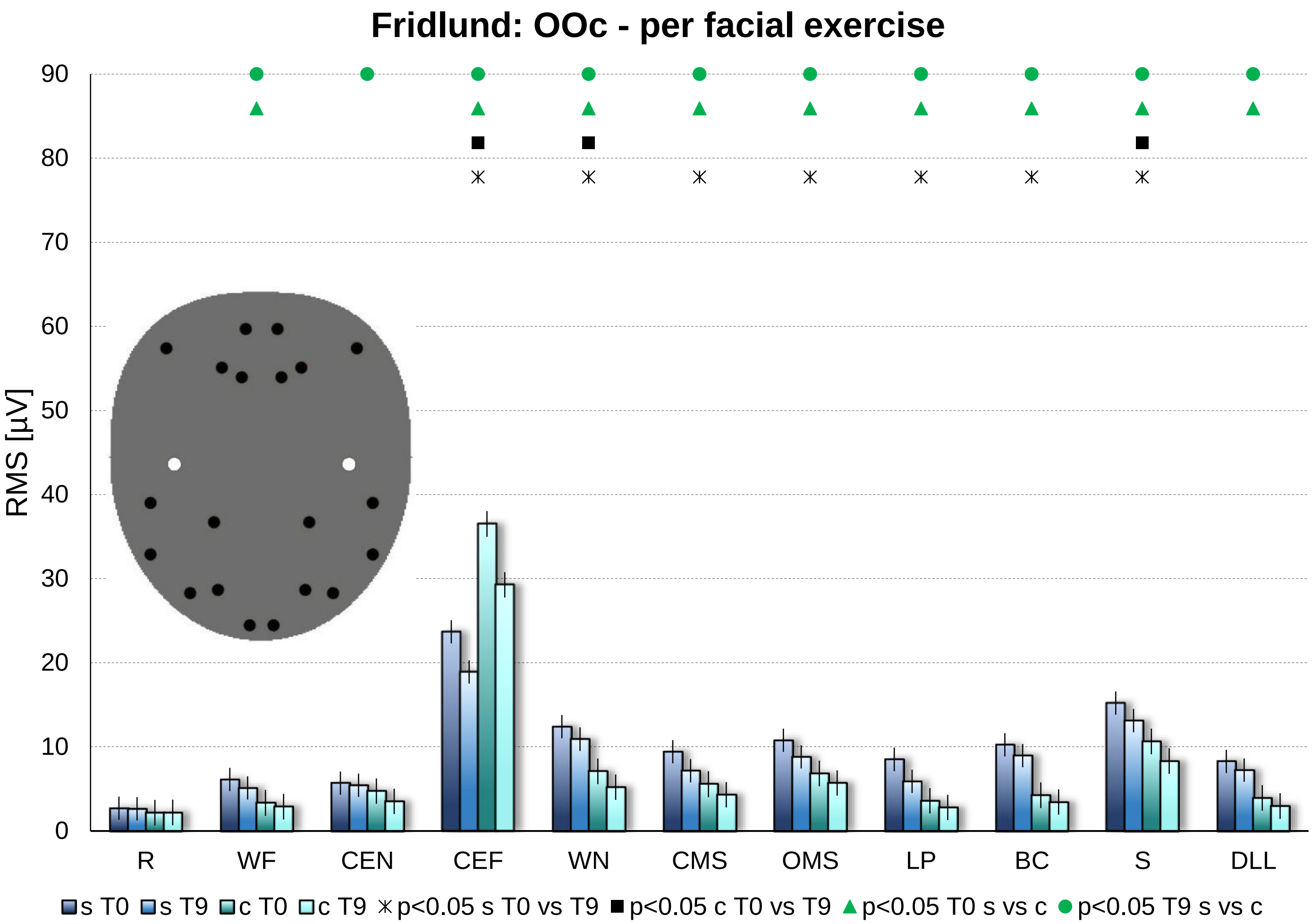
Fridlund: OOc - per facial exercise
RMS [µV]
0
10
20
30
40
50
60
70
80
90
R
WF
CEN
CEF
WN
CMS
OMS
LP
BC
S
DLL
s T0
s T9
c T0
c T9
p<0.05 s T0 vs T9
p<0.05 c T0 vs T9
p<0.05 T0 s vs c
p<0.05 T9 s vs c

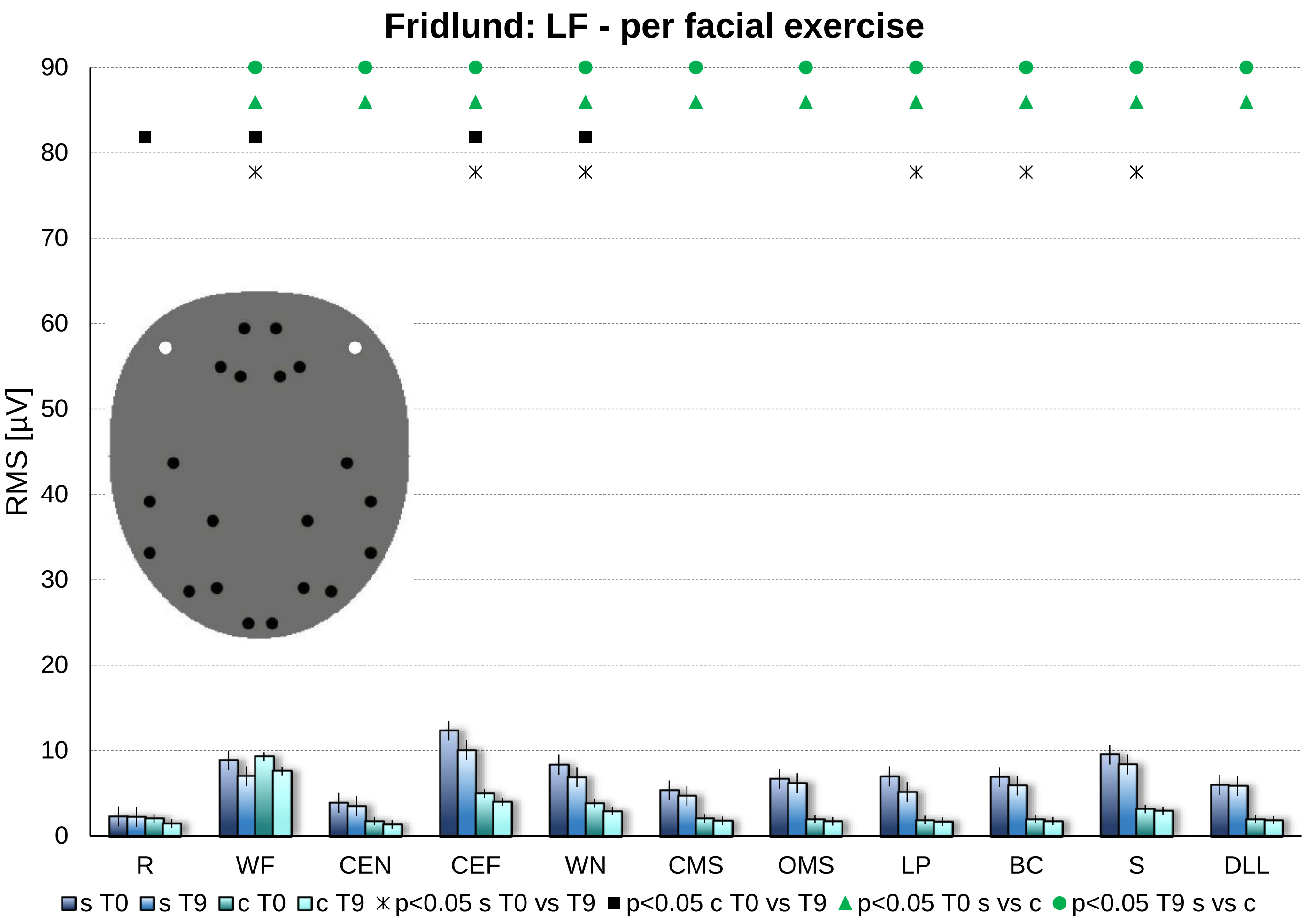
Fridlund: LF - per facial exercise
RMS [µV]
0
10
20
30
40
50
60
70
80
90
R
WF
CEN
CEF
WN
CMS
OMS
LP
BC
S
DLL
s T0
s T9
c T0
c T9
p<0.05 s T0 vs T9
p<0.05 c T0 vs T9
p<0.05 T0 s vs c
p<0.05 T9 s vs c

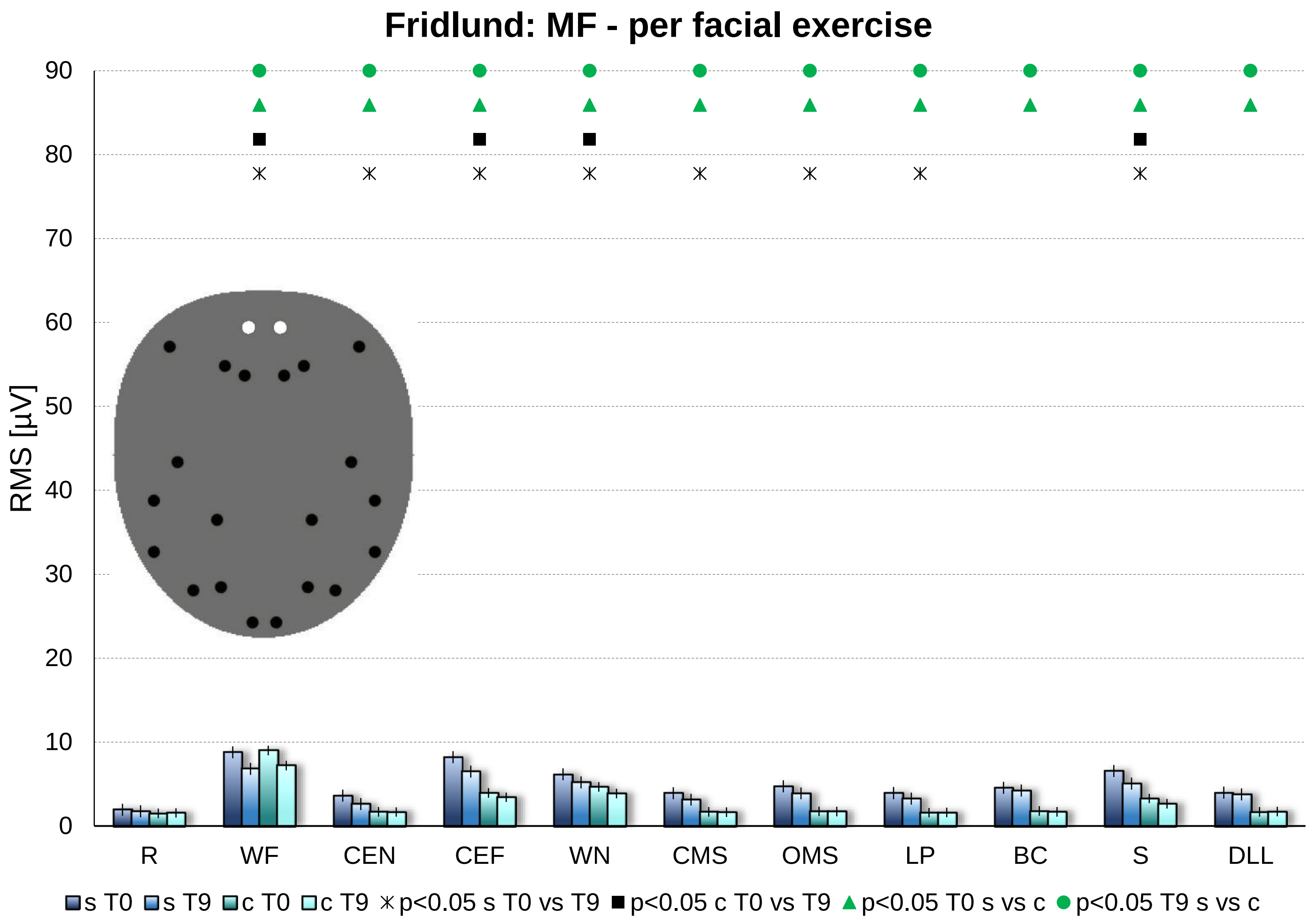

Fridlund: MF - per facial exercise
RMS [µV]
0 10 20 30 40 50 60 70 80 90
R WF CEN CEF WN CMS OMS LP BC S DLL
s T0 s T9 c T0 c T9 p<0.05 s T0 vs T9 p<0.05 c T0 vs T9 p<0.05 T0 s vs c p<0.05 T9 s vs c

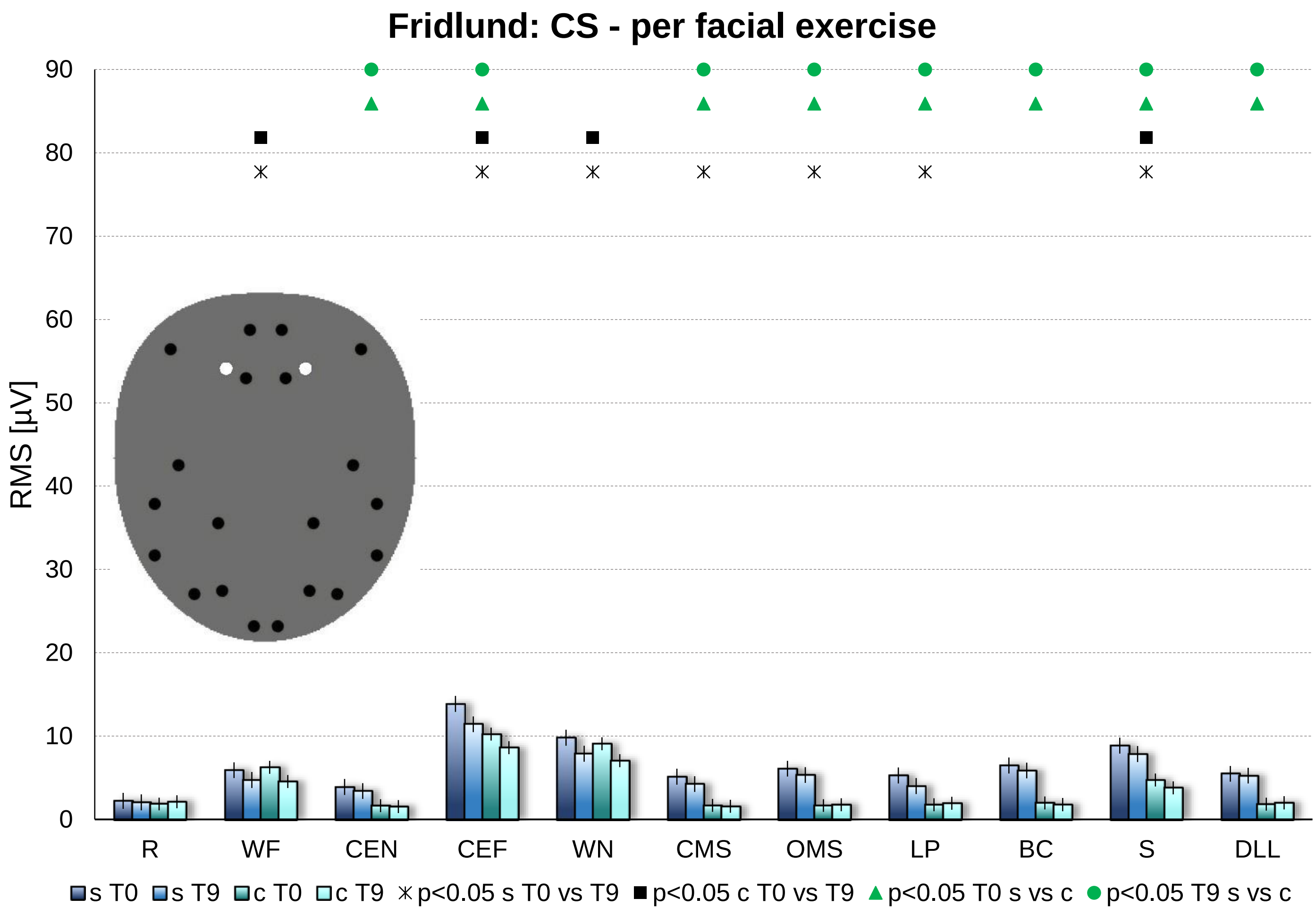

Fridlund: CS - per facial exercise
RMS [µV]
0 10 20 30 40 50 60 70 80 90
R WF CEN CEF WN CMS OMS LP BC S DLL
s T0 s T9 c T0 c T9 p<0.05 s T0 vs T9 p<0.05 c T0 vs T9 p<0.05 T0 s vs c p<0.05 T9 s vs c

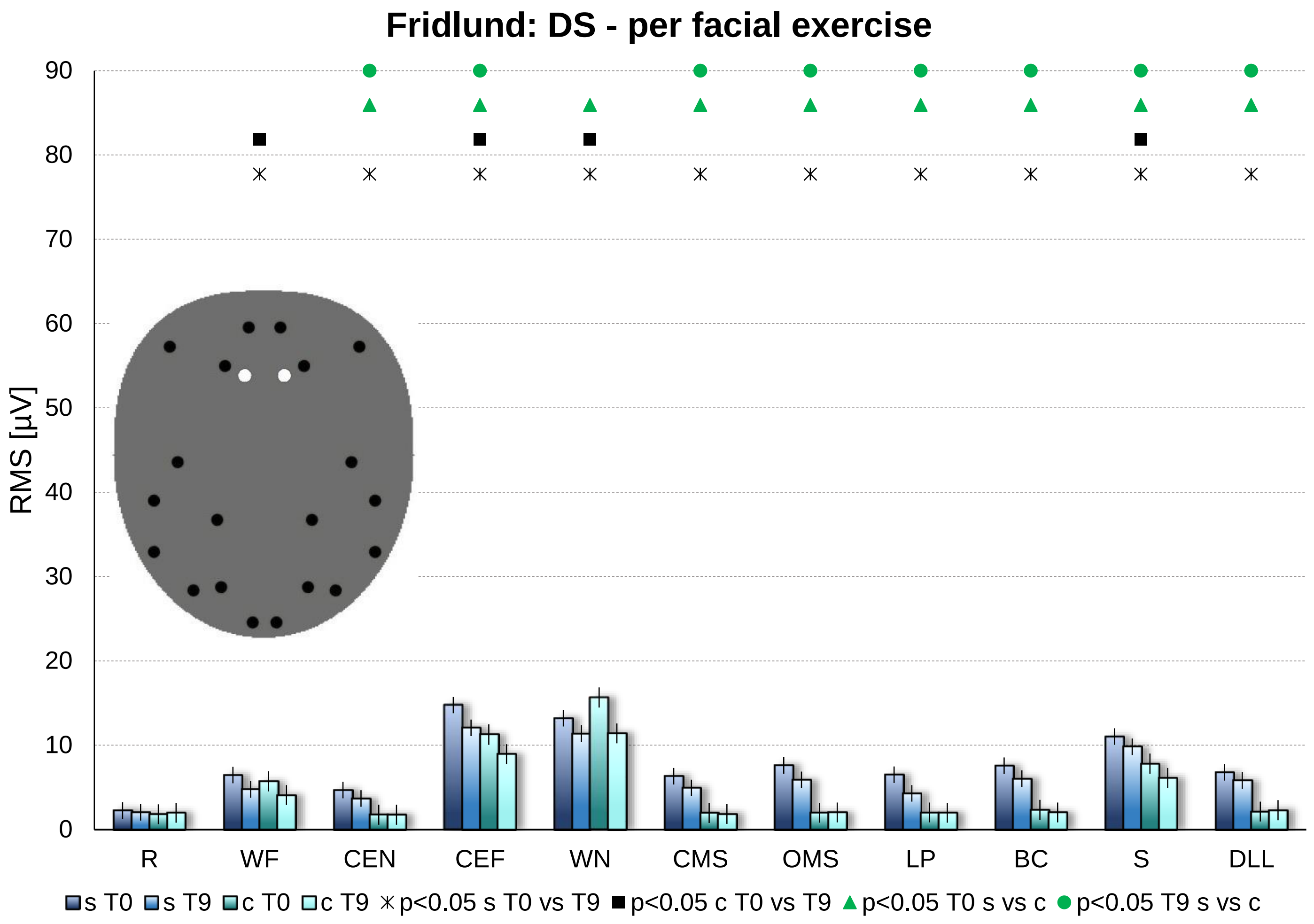

Fridlund: DS - per facial exercise
RMS [µV]
90
80
70
60
50
40
30
20
10
0
R
WF
CEN
CEF
WN
CMS
OMS
LP
BC
S
DLL
s T0
s T9
c T0
c T9
p<0.05 s T0 vs T9
p<0.05 c T0 vs T9
p<0.05 T0 s vs c
p<0.05 T9 s vs c

Electrode schemes
KURAMOTO electrodes E1-E24

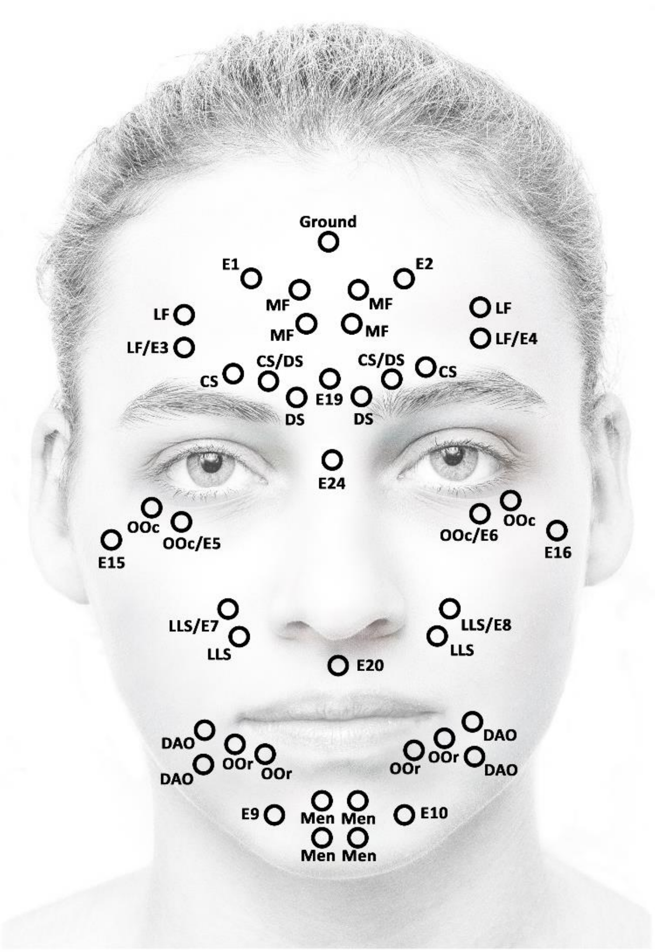

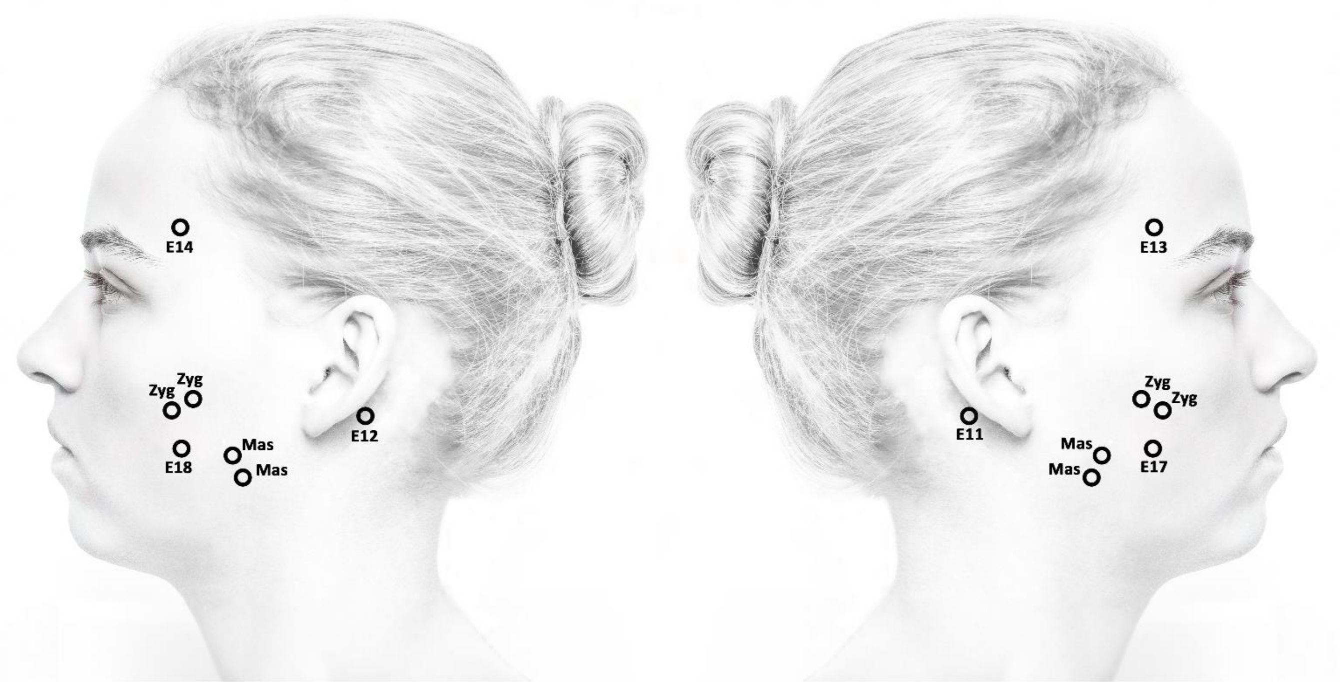

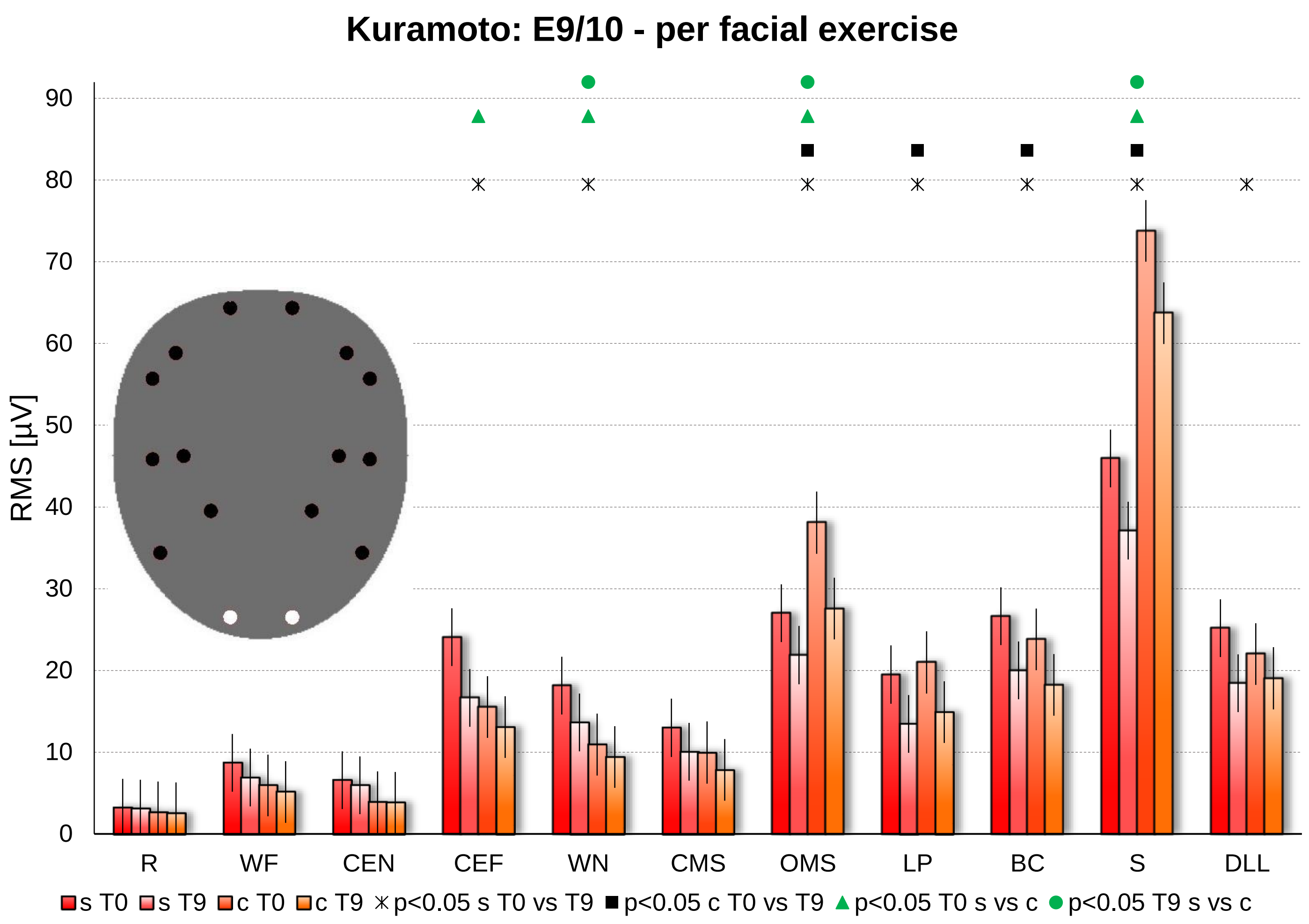

Kuramoto: E9/10 - per facial exercise
RMS [µV]
90
80
70
60
50
40
30
20
10
0
R
WF
CEN
CEF
WN
CMS
OMS
LP
BC
S
DLL
s T0
s T9
c T0
c T9
p<0.05 s T0 vs T9
p<0.05 c T0 vs T9
p<0.05 T0 s vs c
p<0.05 T9 s vs c

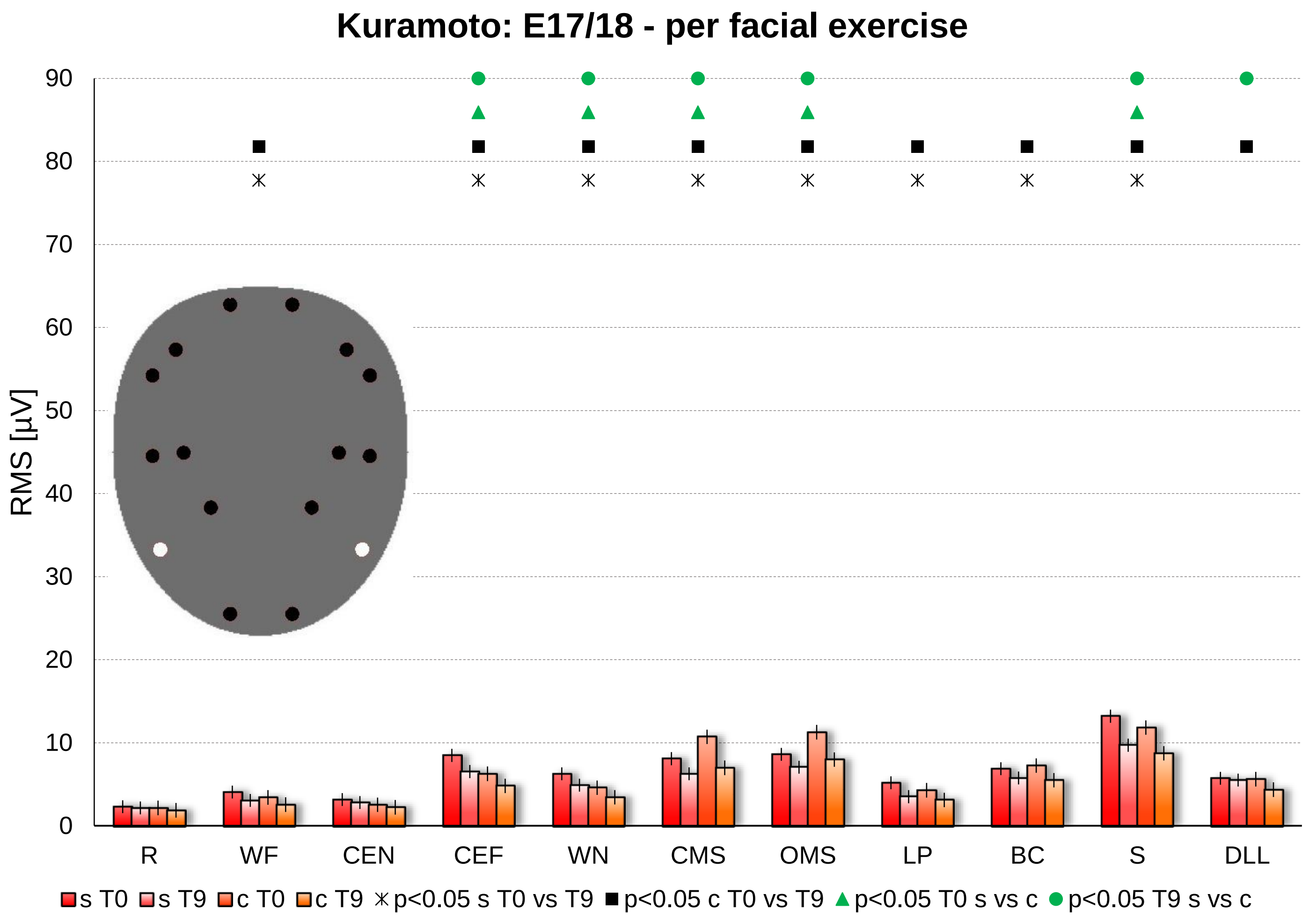

Kuramoto: E17/18 - per facial exercise
RMS [µV]
90
80
70
60
50
40
30
20
10
0
R
WF
CEN
CEF
WN
CMS
OMS
LP
BC
S
DLL
s T0
s T9
c T0
c T9
p<0.05 s T0 vs T9
p<0.05 c T0 vs T9
p<0.05 T0 s vs c
p<0.05 T9 s vs c

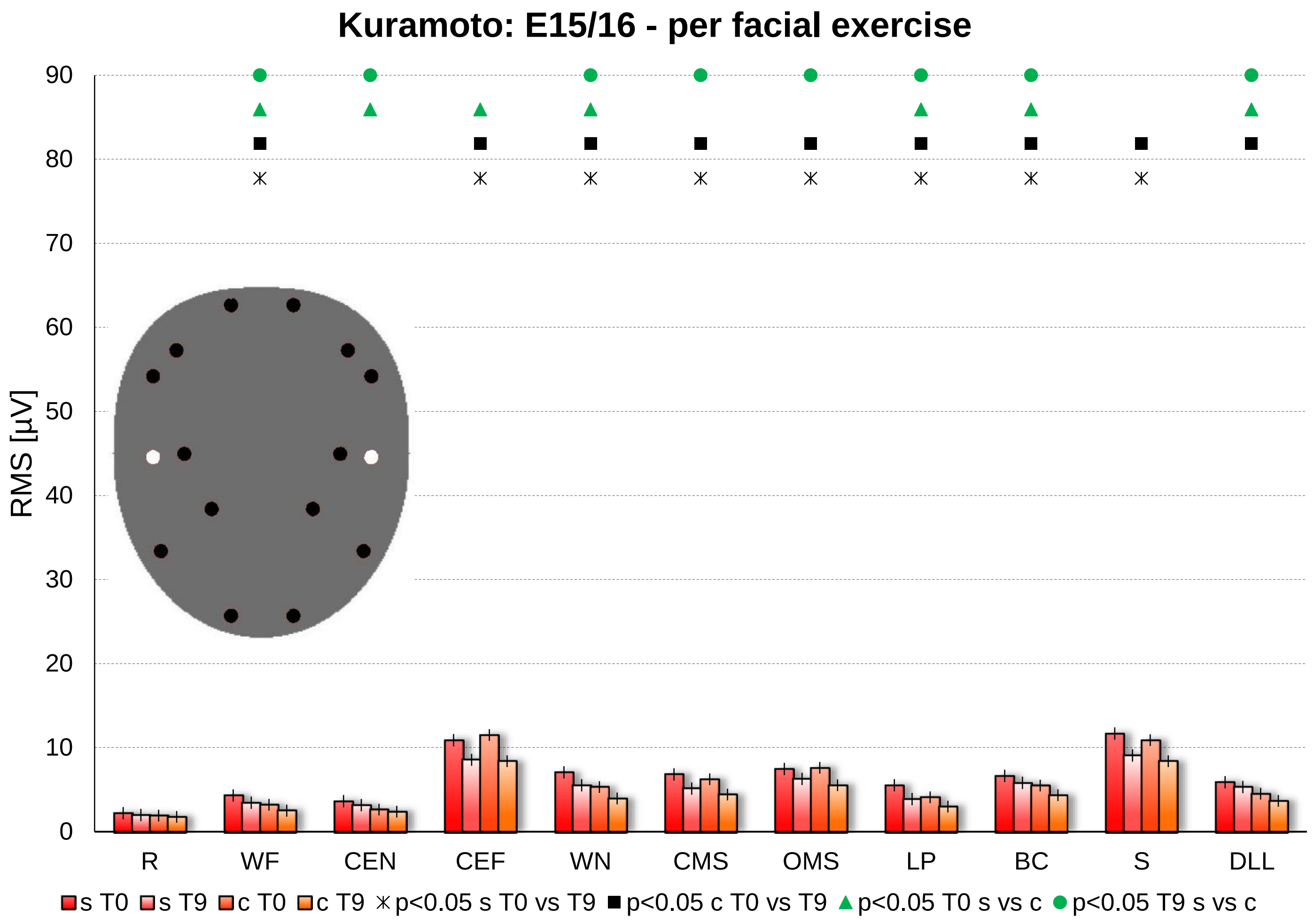
Kuramoto: E15/16 - per facial exercise
RMS [µV]
0
10
20
30
40
50
60
70
80
90
R
WF
CEN
CEF
WN
CMS
OMS
LP
BC
S
DLL
s T0
s T9
c T0
c T9
p<0.05 s T0 vs T9
p<0.05 c T0 vs T9
p<0.05 T0 s vs c
p<0.05 T9 s vs c

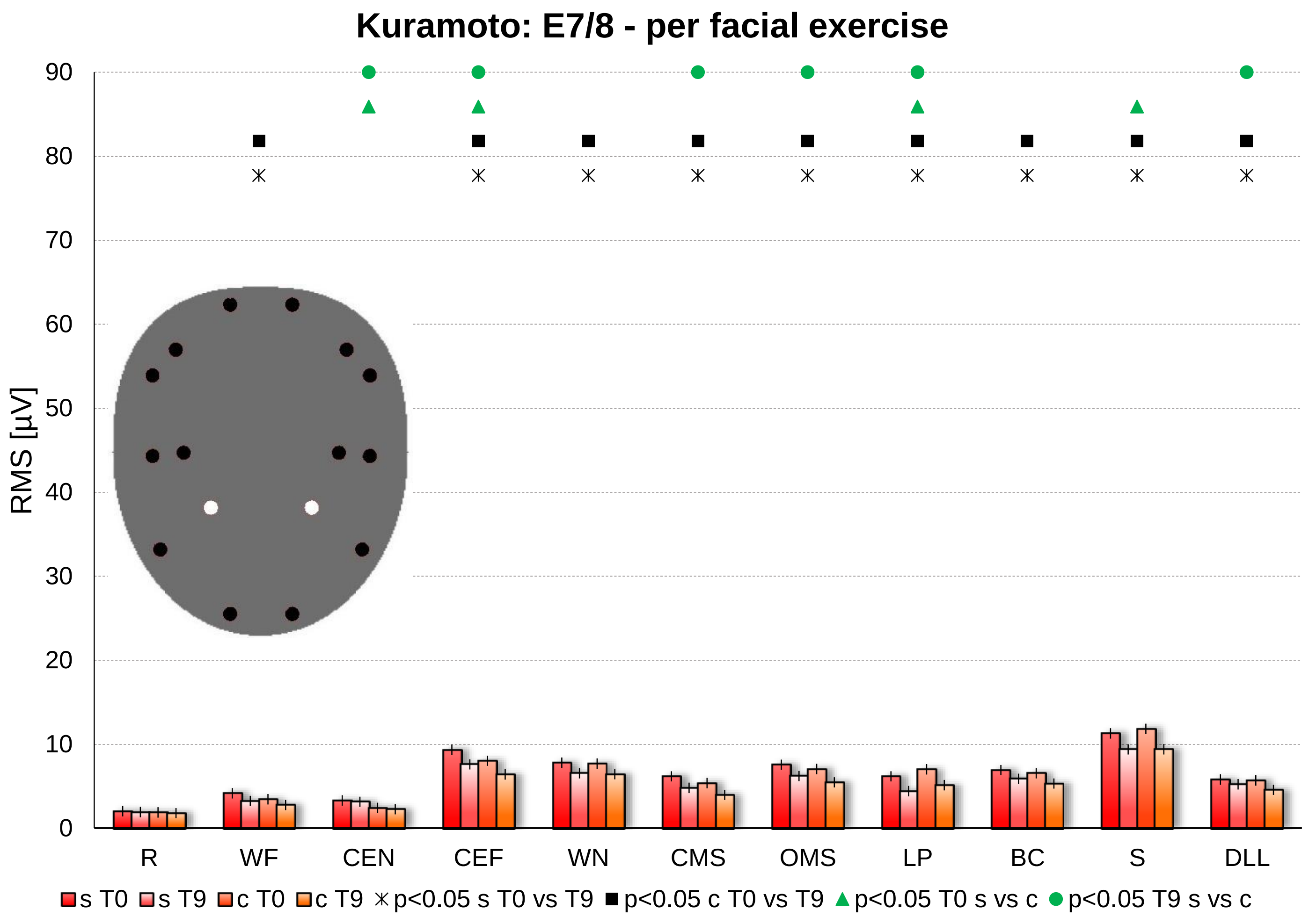
Kuramoto: E7/8 - per facial exercise
RMS [µV]
0
10
20
30
40
50
60
70
80
90
R
WF
CEN
CEF
WN
CMS
OMS
LP
BC
S
DLL
s T0
s T9
c T0
c T9
p<0.05 s T0 vs T9
p<0.05 c T0 vs T9
p<0.05 T0 s vs c
p<0.05 T9 s vs c

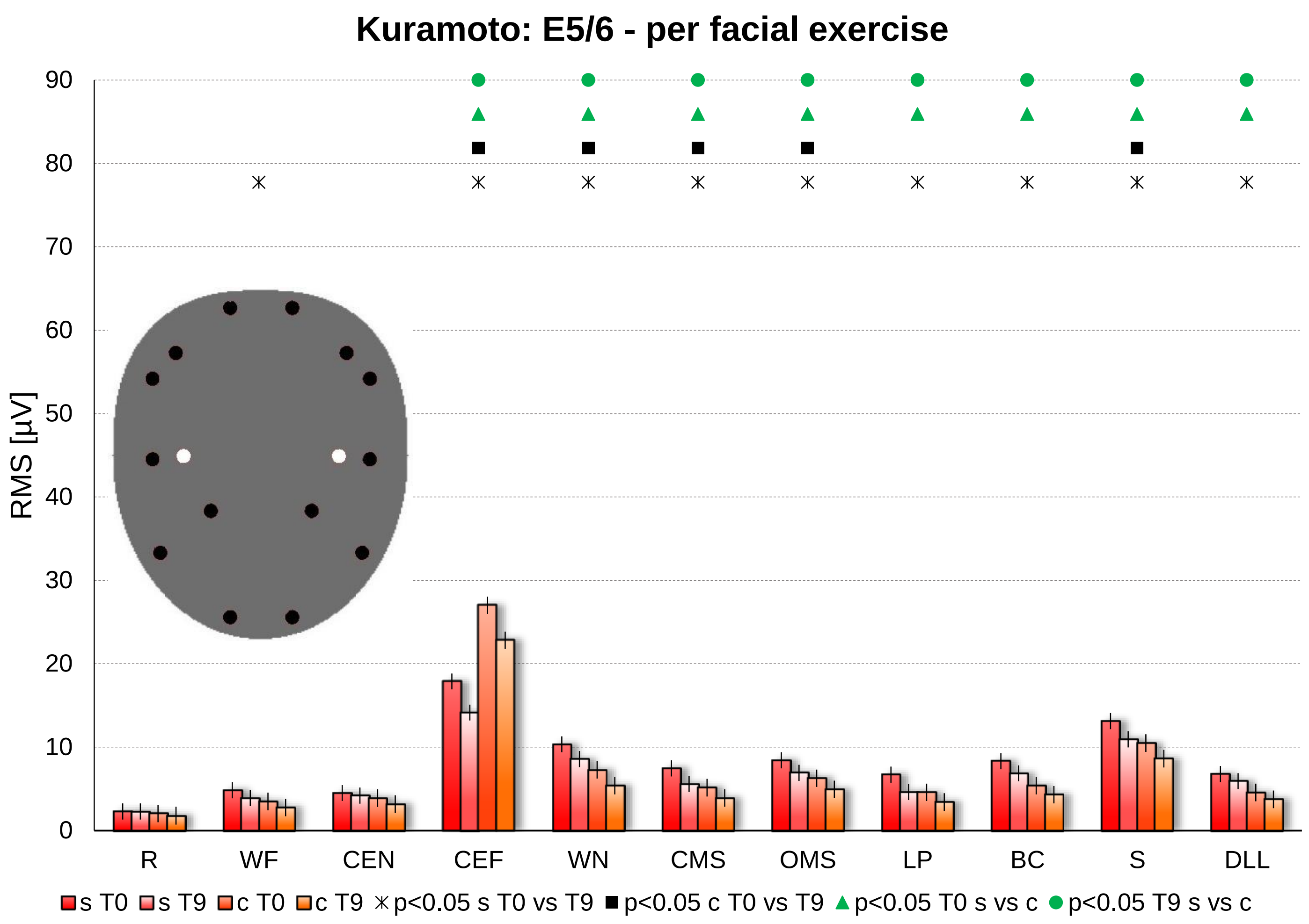

Kuramoto: E5/6 - per facial exercise
RMS [µV]
0
10
20
30
40
50
60
70
80
90
R
WF
CEN
CEF
WN
CMS
OMS
LP
BC
S
DLL
s T0
s T9
c T0
c T9
p<0.05 s T0 vs T9
p<0.05 c T0 vs T9
p<0.05 T0 s vs c
p<0.05 T9 s vs c

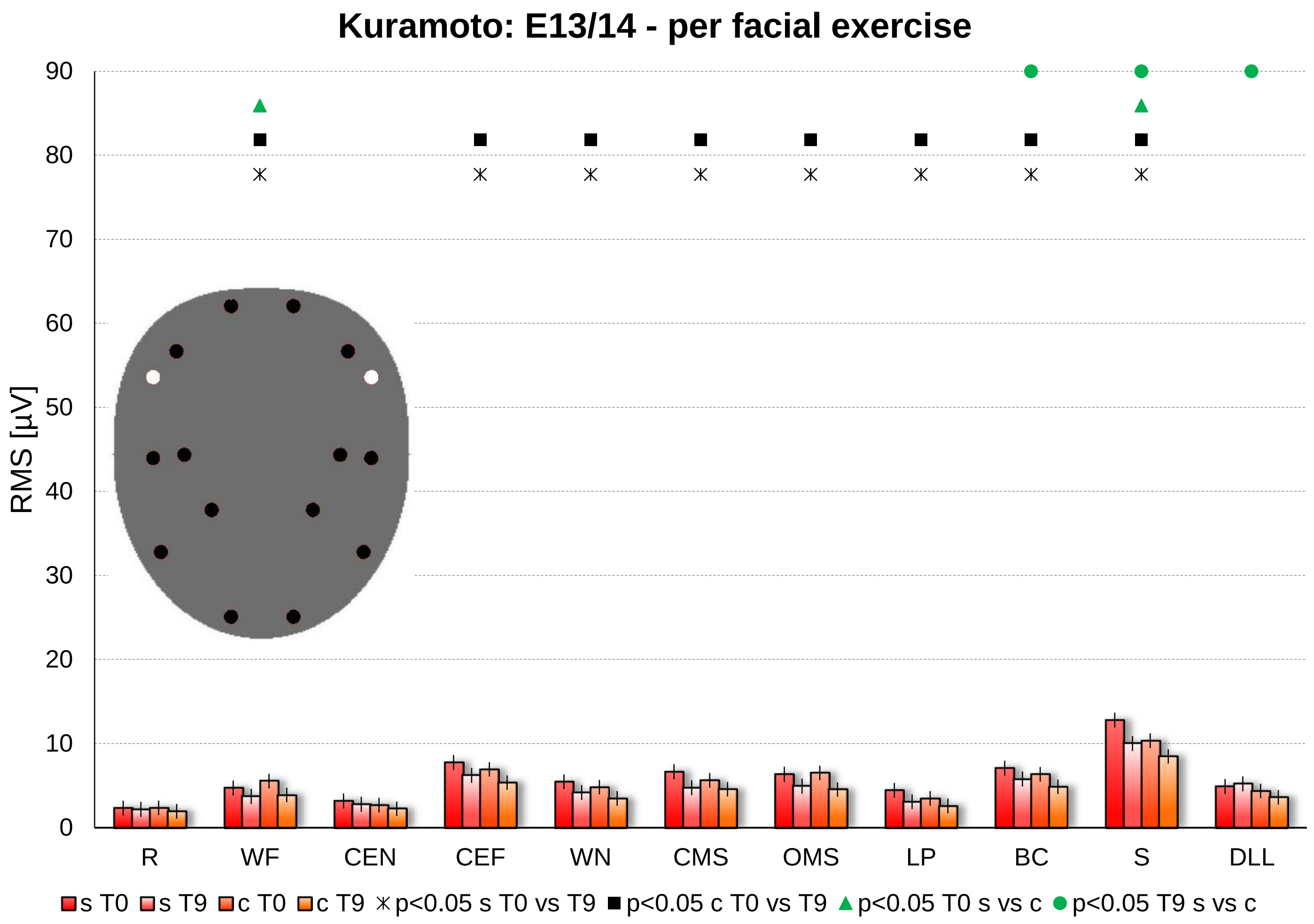

Kuramoto: E13/14 - per facial exercise
RMS [µV]
0
10
20
30
40
50
60
70
80
90
R
WF
CEN
CEF
WN
CMS
OMS
LP
BC
S
DLL
s T0
s T9
c T0
c T9
p<0.05 s T0 vs T9
p<0.05 c T0 vs T9
p<0.05 T0 s vs c
p<0.05 T9 s vs c

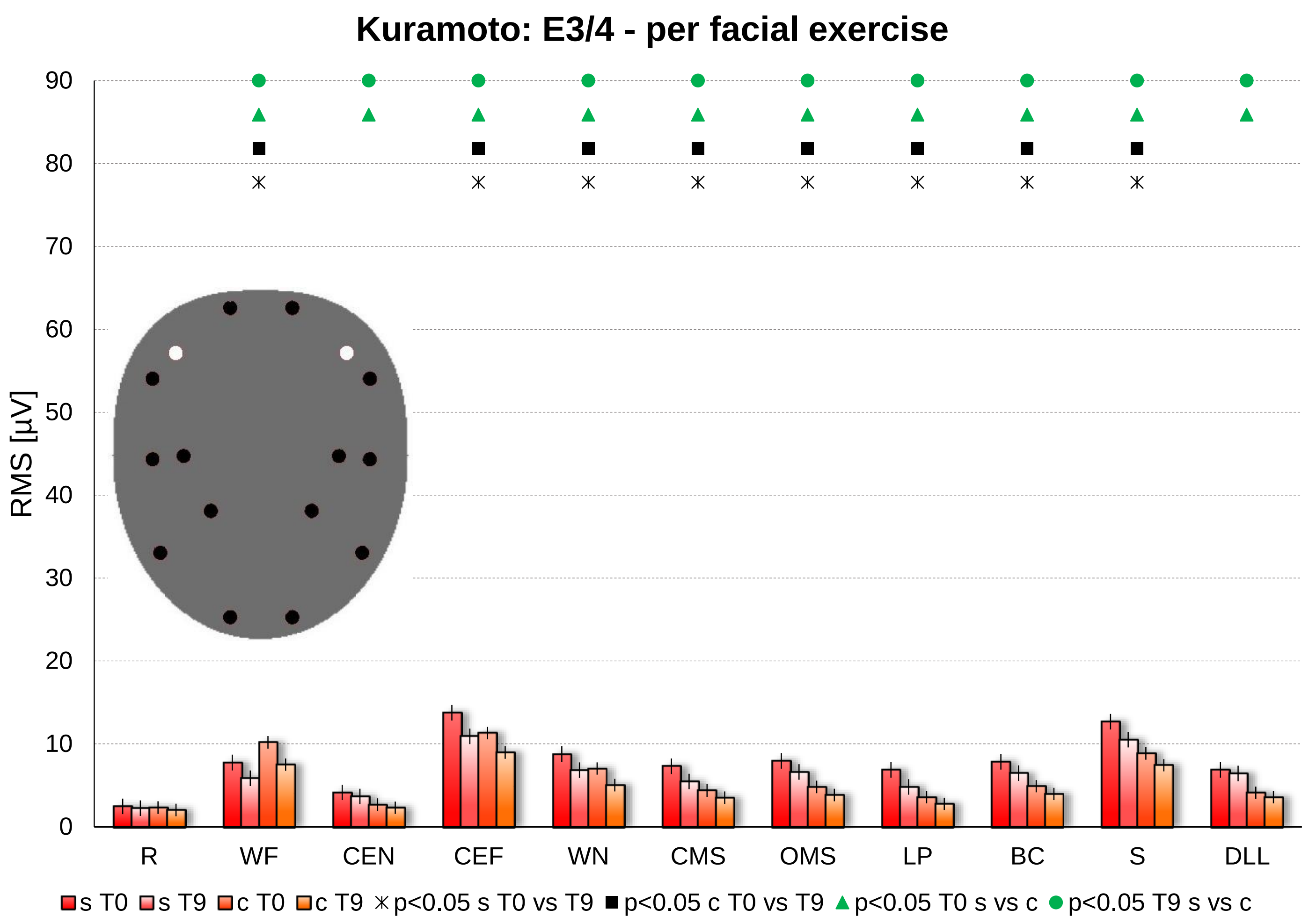

Kuramoto: E3/4 - per facial exercise
RMS [µV]
90
80
70
60
50
40
30
20
10
0
R
WF
CEN
CEF
WN
CMS
OMS
LP
BC
S
DLL
s T0
s T9
c T0
c T9
p<0.05 s T0 vs T9
p<0.05 c T0 vs T9
p<0.05 T0 s vs c
p<0.05 T9 s vs c

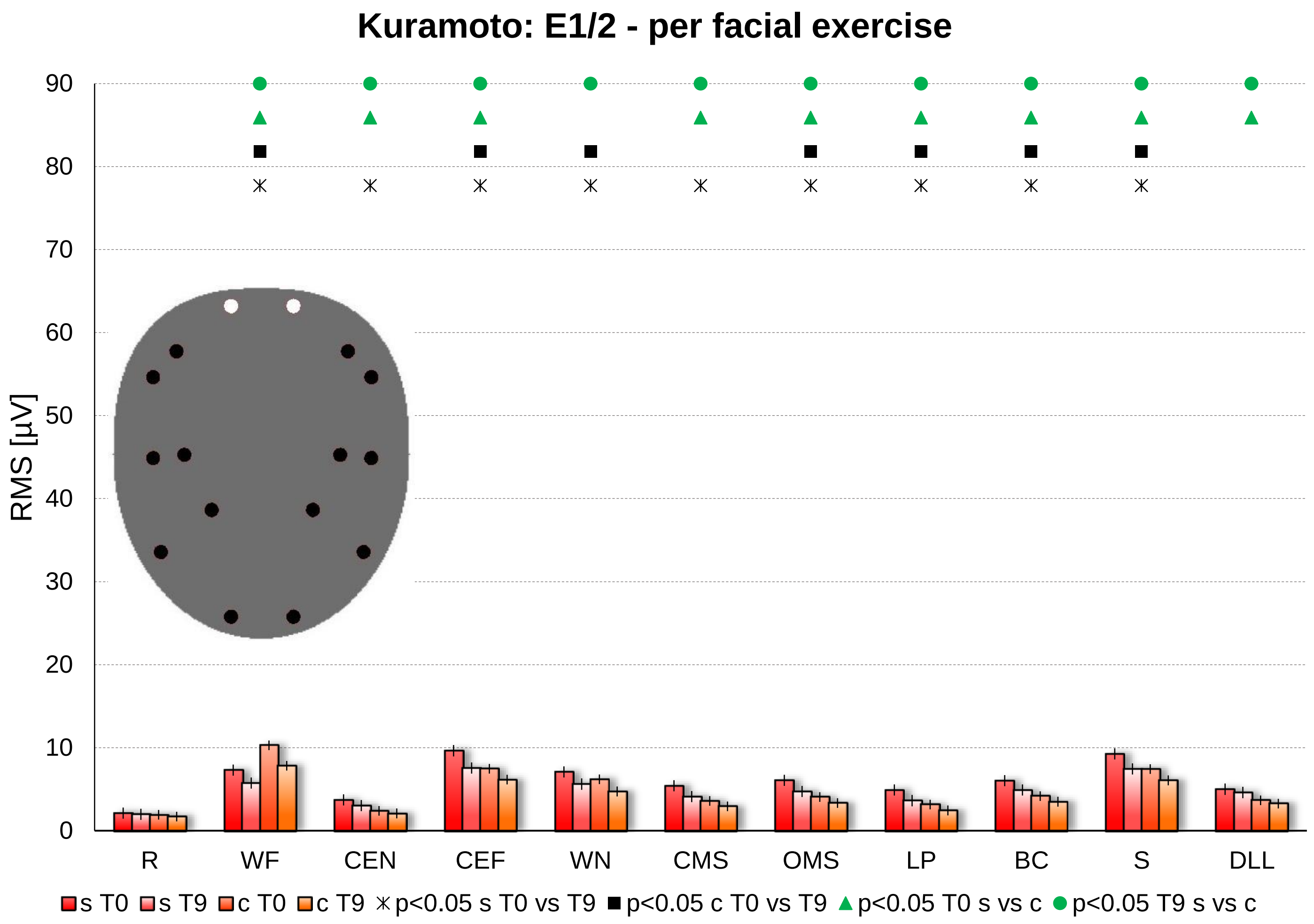

Kuramoto: E1/2 - per facial exercise
RMS [µV]
90
80
70
60
50
40
30
20
10
0
R
WF
CEN
CEF
WN
CMS
OMS
LP
BC
S
DLL
s T0
s T9
c T0
c T9
p<0.05 s T0 vs T9
p<0.05 c T0 vs T9
p<0.05 T0 s vs c
p<0.05 T9 s vs c
