# Supplementary material for: Synchronous surface electromyography as objective method to evaluate the outcome of a biofeedback training in patients with facial synkinesis
Source: Sci Rep. 2025 May 19;15:17335. doi: 10.1038/s41598-025-01278-7 (PMC12089512; doi:10.1038/s41598-025-01278-7)
Supplement: Supplementary file 4 — Supplementary Material 4 [file 41598_2025_1278_MOESM4_ESM.docx]

**Synchronous surface electromyography as objective method to evaluate the outcome of a biofeedback training in patients with facial synkinesis**

Richard Schneider, Maren Schramm, Paul F. Funk, Gerd Fabian Volk, Christoph Anders, Orlando Guntinas-Lichius

**Supplementary Tables**

Supplementary Table S1

Supplementary Table S2

Supplementary Table S3

Supplementary Table S4

**Supplement Table S1.** **Fridlund: Comparison of T0 versus T9 for the each muscle activity and for each functional facial task separately.***

| **Supplement Table S1.** Fridlund: Comparison of T0 versus T9 for the each muscle activity and for each functional facial task separately**.*** | | | | | | | | | | | | |
| --- | --- | --- | --- | --- | --- | --- | --- | --- | --- | --- | --- | --- |
| **Muscle** | **Statistics** | **R** | **WF** | **CEN** | **CEF** | **WN** | **CMS** | **OMS** | **LP** | **BC** | **S** | **DLL** |
| **DAO** | F-value, synkinetic side |  |  |  | 13.86 | 17.85 | 7.60 |  | 41.2 | 23.16 | 31.13 | 13.42 |
|  | p-value, synkinetic side |  |  |  | <0.001 | <0.001 | 0.006 |  | <0.001 | <0.001 | <0.001 | <0.001 |
|  | F-value, contralateral side |  |  |  |  |  |  | 6.28 | 18.18 |  | 22.29 | 45.89 |
|  | p-value, contralateral side |  |  |  |  |  |  | 0.012 | <0.001 |  | <0.001 | <0.001 |
| **OOr** | F-value, synkinetic side |  |  |  |  |  |  | 10.86 | 18.54 | 5.83 | 8.76 |  |
|  | p-value, synkinetic side |  |  |  |  |  |  | 0.001 | <0.001 | 0.016 | 0.003 |  |
|  | F-value, contralateral side |  |  |  |  |  |  | 32.76 | 28.58 |  | 106.09 |  |
|  | p-value, contralateral side |  |  |  |  |  |  | <0.001 | <0.001 |  | <0.001 |  |
| **Men** | F-value, synkinetic side |  |  | 0.180 |  |  |  | 1.52 | 19.84 | 34.08 |  | 13.66 |
|  | p-value, synkinetic side |  |  | <0.001 |  |  |  |  | <0.001 | <0.001 |  | <0.001 |
|  | F-value, contralateral side |  |  |  |  |  |  | 11.40 | 18.96 | 26.18 | 32.76 | 18.48 |
|  | p-value, contralateral side |  |  |  |  |  |  | 0.001 | <0.001 | <0.001 | <0.001 | <0.001 |
| **Mas** | F-value, synkinetic side |  |  |  | 4.25 |  | 6.19 | 5.90 |  |  | 69.84 |  |
|  | p-value, synkinetic side |  |  |  | 0.039 |  | 0.013 | 0.015 |  |  | <0.001 |  |
|  | F-value, contralateral side |  |  |  |  |  |  |  |  |  | 48.85 |  |
|  | p-value, contralateral side |  |  |  |  |  |  |  |  |  | <0.001 |  |
| **Zyg** | F-value, synkinetic side |  |  |  | 19.25 | 8.61 | 31.91 | 21.97 | 5.37 |  | 24.47 |  |
|  | p-value, synkinetic side |  |  |  | <0.001 | 0.003 | <0.001 | <0.001 | 0.021 |  | <0.001 |  |
|  | F-value, contralateral side |  |  |  |  |  | 59.14 | 56.64 |  | 10.43 | 66.62 |  |
|  | p-value, contralateral side |  |  |  |  |  | <0.001 | <0.001 |  | 0.001 | <0.001 |  |
| **LLS** | F-value, synkinetic side |  |  |  | 22.14 | 15.13 | 17.73 | 17.48 | 21.17 | 11.28 | 11.63 |  |
|  | p-value, synkinetic side |  |  |  | <0.001 | <0.001 | <0.001 | <0.001 | <0.001 | 0.001 | 0.001 |  |
|  | F-value, contralateral side |  |  |  | 5.87 | 39.92 | 10.45 | 35.14 | 39.65 | 6.86 | 149.39 | 6.25 |
|  | p-value, contralateral side |  |  |  | 0.015 | <0.001 | 0.001 | <0.001 | <0.001 | 0.009 | <0.001 | 0.012 |
| **OOc** | F-value, synkinetic side |  |  |  | 65.51 | 6.22 | 15.03 | 11.51 | 19.29 | 4.75 | 12.44 |  |
|  | p-value, synkinetic side |  |  |  | <0.001 | 0.013 | <0.001 | 0.001 | <0.001 | 0.029 | <0.001 |  |
|  | F-value, contralateral side |  |  |  | 70.19 | 4.70 |  |  |  |  | 7.20 |  |
|  | p-value, contralateral side |  |  |  | <0.001 | 0.030 |  |  |  |  | 0.007 |  |
| **LF** | F-value, synkinetic side |  | 15.52 |  | 22.89 | 9.59 |  |  | 14.50 | 4.29 | 5.99 |  |
|  | p-value, synkinetic side |  | <0.001 |  | <0.001 | 0.002 |  |  | <0.001 | 0.038 | 0.014 |  |
|  | F-value, contralateral side | 5.21 | 47.24 |  | 14.59 | 14.67 |  |  |  |  |  |  |
|  | p-value, contralateral side | 0.023 | <0.001 |  | <0.001 | <0.001 |  |  |  |  |  |  |
| **MF** | F-value, synkinetic side |  | 42.43 | 10.21 | 30.64 | 9.63 | 6.49 | 7.71 | 4.51 |  | 24.76 |  |
|  | p-value, synkinetic side |  | <0.001 | 0.001 | <0.001 | 0.002 | 0.011 | 0.006 | 0.034 |  | <0.001 |  |
|  | F-value, contralateral side |  | 49.51 |  | 3.89 | 9.34 |  |  |  |  | 5.78 |  |
|  | p-value, contralateral side |  | <0.001 |  | 0.049 | 0.002 |  |  |  |  | 0.016 |  |
| **CS** | F-value, synkinetic side |  | 10.31 |  | 43.12 | 26.51 | 5.72 | 4.25 | 11.54 |  | 7.71 |  |
|  | p-value, synkinetic side |  | 0.001 |  | <0.001 | <0.001 | 0.017 | 0.039 | 0.001 |  | 0.006 |  |
|  | F-value, contralateral side |  | 16.37 |  | 14.96 | 13.12 |  |  |  |  | 4.91 |  |
|  | p-value, contralateral side |  | <0.001 |  | <0.001 | <0.001 |  |  |  |  | 0.027 |  |
| **DS** | F-value, synkinetic side |  | 12.74 | 4.38 | 32.47 | 15.13 | 8.58 | 13.36 | 21.74 | 10.34 | 6.37 | 4.16 |
|  | p-value, synkinetic side |  | <0.001 | 0.036 | <0.001 | <0.001 | 0.003 | <0.001 | <0.001 | 0.001 | 0.012 | 0.042 |
|  | F-value, contralateral side |  | 7.42 |  | 15.06 | 51.16 |  |  |  |  | 8.24 |  |
|  | p-value, contralateral side |  | 0.006 |  | <0.001 | <0.001 |  |  |  |  | 0.004 |  |

*empty grey fields = no significant difference (p>0.05); R = at rest; WF = Wrinkling of the forehead; CEN = Closing the eyes normally; CEF = Closing the eyes forcefully; WN = Wrinkling of the nose; CMS = Closed mouth smiling; OMS = Open mouth smiling; LP = Lip puckering; BC = Blowing-out the cheeks; S = Snarling; DLL = Depressing lower lip; MF = frontal muscle. medial part; LF = frontal muscle. lateral part; CS = corrugator supercilii muscle; DS = depressor supercilii muscle; OOc = orbibularis oculi muscle; Zyg = zygomatic muscle; LLS = levator labii superioris muscle; Mass = masseter muscle (not innervated by facial nerve. control muscle; OOr = orbicularis oris muscle; DAO = depressor anguli oris muscle; Men = mentalis muscle.

**Supplement Table S2. Fridlund: Comparison of synkinetic versus contralateral side for the each muscle activity and for each functional facial task separately.***

| **Supplement Table 2.** Fridlund: Comparison of synkinetic versus contralateral side for the each muscle activity and for each functional facial task separately**.*** | | | | | | | | | | | | | |
| --- | --- | --- | --- | --- | --- | --- | --- | --- | --- | --- | --- | --- | --- |
| **Muscle** | **Statistics** | **R** | **WF** | **CEN** | **CEF** | **WN** | **CMS** | **OMS** | **LP** | **BC** | **S** | **DLL** |  |
| **DAO** | F-value, T0 |  | 24.23 | 25.21 | 137.69 | 49.64 | 33.32 | 11.67 |  | 65.41 |  | 144.96 |  |
|  | p-value, T0 |  | <0.001 | <0.001 | <0.001 | <0.001 | <0.001 | 0.001 |  | <0.001 |  | <0.001 |  |
|  | F-value, T9 |  | 27.27 | 21.94 | 149.46 | 45.46 | 36.63 | 34.52 |  | 49.96 |  | 77.59 |  |
|  | p-value, T9 |  | <0.001 | <0.001 | <0.001 | <0.001 | <0.001 | <0.001 |  | <0.001 |  | <0.001 |  |
| **OOr** | F-value, T0 |  | 8.51 | 14.38 | 85.08 | 38.24 | 8.70 | 10.86 | 71.37 | 4.62 | 218.57 |  |  |
|  | p-value, T0 |  | 0.004 | <0.001 | <0.001 | <0.001 | 0.003 | <0.001 | <0.001 | 0.032 | <0.001 |  |  |
|  | F-value, T9 |  | 7.63 | 12.64 | 89.77 | 33.45 | 11.88 |  | 49.04 | 4.47 | 48.81 |  |  |
|  | p-value, T9 |  | 0.006 | <0.001 | <0.001 | <0.001 | <0.001 |  | <0.001 | 0.035 | <0.001 |  |  |
| **Men** | F-value, T0 |  | 0.84 | 0.03 | 14.15 | 3.87 |  | 10.16 |  | 8.64 | 86.31 | 20.32 |  |
|  | p-value, T0 |  |  |  | <0.001 | 0.049 |  | 0.001 |  | 0.003 | <0.001 | <0.001 |  |
|  | F-value, T9 |  |  |  | 3.91 |  |  |  |  | 6.48 | 21.73 | 10.93 |  |
|  | p-value, T9 |  |  |  | 0.048 |  |  |  |  | 0.011 | <0.001 | <0.001 |  |
| **Mas** | F-value, T0 |  |  |  | 8.61 |  | 5.76 |  |  |  |  |  |  |
|  | p-value, T0 |  |  |  | 0.003 |  | 0.016 |  |  |  |  |  |  |
|  | F-value, T9 |  |  |  | 5.25 |  |  |  |  |  |  | 4.87 |  |
|  | p-value, T9 |  |  |  | 0.022 |  |  |  |  |  |  | 0.027 |  |
| **Zyg** | F-value, T0 |  | 5.90 | 5.97 | 91.16 | 47.58 | 10.34 | 31.42 | 10.09 |  |  | 14.89 |  |
|  | p-value, T0 |  | 0.015 | 0.015 | <0.001 | <0.001 | 0.001 | <0.001 | 0.002 |  |  | <0.001 |  |
|  | F-value, T9 |  |  | 5.09 | 106.81 | 56.61 |  | 13.59 | 5.89 |  | 15.79 | 23.93 |  |
|  | p-value, T9 |  |  | 0.024 | <0.001 | <0.001 |  | <0.001 | 0.015 |  | <0.001 | <0.001 |  |
| **LLS** | F-value, T0 |  | 9.12 | 12.94 | 88.01 | 7.63 | 11.87 |  | 15.34 | 11.26 | 64.02 |  |  |
|  | p-value, T0 |  | 0.003 | <0.001 | <0.001 | 0.006 | 0.001 |  | <0.001 | 0.001 | <0.001 |  |  |
|  | F-value, T9 |  | 5.68 | 11.11 | 69.13 |  | 10.83 | 9.30 | 5.94 | 10.45 |  |  |  |
|  | p-value, T9 |  | 0.017 | 0.001 | <0.001 |  | 0.001 | 0.002 | 0.015 | 0.001 |  |  |  |
| **OOc** | F-value, T0 |  | 7.13 |  | 297.68 | 34.36 | 15.41 | 17.08 | 28.87 | 44.75 | 23.63 | 21.35 |  |
|  | p-value, T0 |  | 0.008 |  | <0.001 | <0.001 | <0.001 | <0.001 | <0.001 | <0.001 | <0.001 | <0.001 |  |
|  | F-value, T9 |  | 7.23 | 6.05 | 221.49 | 61.05 | 13.40 | 17.00 | 16.35 | 55.62 | 41.28 | 31.01 |  |
|  | p-value, T9 |  | 0.007 | 0.014 | <0.001 | <0.001 | <0.001 | <0.001 | <0.001 | <0.001 | <0.001 | <0.001 |  |
| **LF** | F-value, T0 |  | 4.76 | 12.84 | 235.54 | 77.65 | 37.29 |  | 86.12 | 102.03 | 94.73 | 170.88 |  |
|  | p-value, T0 |  | 0.029 | <0.001 | <0.001 | <0.001 | <0.001 |  | <0.001 | <0.001 | <0.001 | <0.001 |  |
|  | F-value, T9 |  | 10.22 | 18.24 | 220.48 | 84.79 | 40.74 |  | 110.70 | 62.40 | 94.09 | 173.35 |  |
|  | p-value, T9 |  | 0.001 | <0.001 | <0.001 | <0.001 | <0.001 |  | <0.001 | <0.001 | <0.001 | <0.001 |  |
| **MF** | F-value, T0 |  | 3.97 | 24.76 | 159.91 | 12.25 | 37.19 | 73.08 | 41.35 | 62.83 | 92.77 | 39.88 |  |
|  | p-value, T0 |  | 0.046 | <0.001 | <0.001 | <0.001 | <0.001 | <0.001 | <0.001 | <0.001 | <0.001 | <0.001 |  |
|  | F-value, T9 |  | 6.08 | 5.49 | 94.27 | 12.79 | 17.39 | 42.35 | 23.07 | 60.91 | 53.78 | 37.55 |  |
|  | p-value, T9 |  | 0.014 | 0.019 | <0.001 | <0.001 | <0.001 | <0.001 | <0.001 | <0.001 | <0.001 | <0.001 |  |
| **CS** | F-value, T0 |  |  | 20.75 | 55.07 |  | 51.39 | 57.57 | 52.94 | 90.54 | 75.85 | 57.96 |  |
|  | p-value, T0 |  |  | <0.001 | <0.001 |  | <0.001 | <0.001 | <0.001 | <0.001 | <0.001 | <0.001 |  |
|  | F-value, T9 |  |  | 18.33 | 52.26 |  | 40.58 | 84.49 | 27.34 | 103.30 | 102.00 | 63.40 |  |
|  | p-value, T9 |  |  | <0.001 | <0.001 |  | <0.001 | <0.001 | <0.001 | <0.001 | <0.001 | <0.001 |  |
| **DS** | F-value, T0 |  |  | 22.88 | 32.79 |  | 51.96 | 89.02 | 55.72 | 76.30 | 26.66 | 60.15 |  |
|  | p-value, T0 |  |  | <0.001 | <0.001 |  | <0.001 | <0.001 | <0.001 | <0.001 | <0.001 | <0.001 |  |
|  | F-value, T9 |  |  | 13.55 | 35.50 |  | 35.67 | 55.62 | 19.23 | 60.12 | 51.03 | 46.51 |  |
|  | p-value, T9 |  |  | <0.001 | <0.001 |  | <0.001 | <0.001 | <0.001 | <0.001 | <0.001 | <0.001 |  |

*empty grey fields = no significant difference (p>0.05); R = at rest; WF = Wrinkling of the forehead; CEN = Closing the eyes normally; CEF = Closing the eyes forcefully; WN = Wrinkling of the nose; CMS = Closed mouth smiling; OMS = Open mouth smiling; LP = Lip puckering; BC = Blowing-out the cheeks; S = Snarling; DLL = Depressing lower lip; MF = frontal muscle. medial part; LF = frontal muscle. lateral part; CS = corrugator supercilii muscle; DS = depressor supercilii muscle; OOc = orbibularis oculi muscle; Zyg = zygomatic muscle; LLS = levator labii superioris muscle; Mass = masseter muscle (not innervated by facial nerve. control muscle; OOr = orbicularis oris muscle; DAO = depressor anguli oris muscle; Men = mentalis muscle.

**Supplement Table S3. Kuramoto: Comparison of T0 versus T9 for the each muscle activity and for each functional facial task separately.***

| **Supplement Table 3.** Kuramoto: Comparison of T0 versus T9 for the each muscle activity and for each functional facial task separately**.*** | | | | | | | | | | | | |
| --- | --- | --- | --- | --- | --- | --- | --- | --- | --- | --- | --- | --- |
| **Muscle** | **Statistics** | **R** | **WF** | **CEN** | **CEF** | **WN** | **CMS** | **OMS** | **LP** | **BC** | **S** | **DLL** |
| **E9/10** | F-value, synkinetic side |  |  |  | 18.74 | 6.86 |  | 8.79 | 12.26 | 14.93 | 26.47 | 15.34 |
|  | p-value, synkinetic side |  |  |  | <0.001 | 0.009 |  | 0.003 | <0.001 | <0.001 | <0.001 | <0.001 |
|  | F-value, contralateral side |  |  |  |  |  |  | 30.57 | 10.33 | 8.89 | 28.75 |  |
|  | p-value, contralateral side |  |  |  |  |  |  | <0.001 | 0.001 | 0.003 | <0.001 |  |
| **E17/E18** | F-value, synkinetic side |  | 7.62 |  | 29.37 | 14.39 | 24.69 | 18.14 | 22.13 | 10.12 | 95.49 |  |
|  | p-value, synkinetic side |  | 0.006 |  | <0.001 | <0.001 | <0.001 | <0.001 | <0.001 | 0.001 | <0.001 |  |
|  | F-value, contralateral side |  | 4.25 |  | 12.46 | 7.96 | 80.02 | 61.21 | 7.98 | 17.92 | 57.38 | 9.15 |
|  | p-value, contralateral side |  | 0.039 |  | <0.001 | 0.005 | <0.001 | <0.001 | 0.005 | <0.001 | <0.001 | 0.003 |
| **E15/16** | F-value, synkinetic side |  | 8.47 |  | 65.31 | 27.16 | 33.17 | 15.71 | 32.30 | 7.90 | 79.09 |  |
|  | p-value, synkinetic side |  | 0.004 |  | <0.001 | <0.001 | <0.001 | <0.001 | <0.001 | 0.005 | <0.001 |  |
|  | F-value, contralateral side |  | 4.01 |  | 81.82 | 14.99 | 27.08 | 35.00 | 10.30 | 11.16 | 52.45 | 6.32 |
|  | p-value, contralateral side |  | 0.045 |  | <0.001 | <0.001 | <0.001 | <0.001 | 0.001 | 0.001 | <0.001 | 0.012 |
| **E7/8** | F-value, synkinetic side |  | 15.64 |  | 54.83 | 28.49 | 34.48 | 34.12 | 56.68 | 19.52 | 67.34 | 6.05 |
|  | p-value, synkinetic side |  | <0.001 |  | <0.001 | <0.001 | <0.001 | <0.001 | <0.001 | <0.001 | <0.001 | 0.014 |
|  | F-value, contralateral side |  | 6.40 |  | 36.77 | 21.67 | 26.90 | 34.54 | 49.29 | 20.65 | 80.26 | 17.56 |
|  | p-value, contralateral side |  | 0.011 |  | <0.001 | <0.001 | <0.001 | <0.001 | <0.001 | <0.001 | <0.001 | <0.001 |
| **E5/6** | F-value, synkinetic side |  | 4.93 |  | 76.21 | 17.27 | 19.69 | 12.21 | 24.12 | 11.77 | 25.73 | 3.94 |
|  | p-value, synkinetic side |  | 0.027 |  | <0.001 | <0.001 | <0.001 | <0.001 | <0.001 | 0.001 | <0.001 | 0.047 |
|  | F-value, contralateral side |  |  |  | 47.27 | 9.69 | 4.19 | 4.74 |  |  | 9.40 |  |
|  | p-value, contralateral side |  |  |  | <0.001 | 0.002 | 0.041 | 0.030 |  |  | 0.002 |  |
| **E13/E14** | F-value, synkinetic side |  | 5.07 |  | 11.75 | 7.91 | 17.44 | 9.66 | 9.27 | 8.51 | 38.45 |  |
|  | p-value, synkinetic side |  | 0.024 |  | 0.001 | 0.005 | <0.001 | 0.002 | 0.002 | 0.004 | <0.001 |  |
|  | F-value, contralateral side |  | 13.59 |  | 12.40 | 8.93 | 5.26 | 19.12 | 4.05 | 11.23 | 17.00 |  |
|  | p-value, contralateral side |  | <0.001 |  | <0.001 | 0.003 | 0.022 | <0.001 | 0.044 | 0.001 | <0.001 |  |
| **E3/4** | F-value, synkinetic side |  | 22.00 |  | 50.65 | 23.86 | 21.24 | 11.37 | 26.81 | 11.38 | 29.42 |  |
|  | p-value, synkinetic side |  | <0.001 |  | <0.001 | <0.001 | <0.001 | 0.001 | <0.001 | 0.001 | <0.001 |  |
|  | F-value, contralateral side |  | 43.22 |  | 33.32 | 24.90 | 4.63 | 5.67 | 3.99 | 5.51 | 12.65 |  |
|  | p-value, contralateral side |  | <0.001 |  | <0.001 | <0.001 | 0.032 | 0.017 | 0.046 | 0.019 | <0.001 |  |
| **E1/2** | F-value, synkinetic side |  | 29.13 |  | 51.74 | 25.34 | 19.72 | 20.89 | 19.99 | 14.74 | 38.88 |  |
|  | p-value, synkinetic side |  | <0.001 |  | <0.001 | <0.001 | <0.001 | <0.001 | <0.001 | <0.001 | <0.001 |  |
|  | F-value, contralateral side |  | 56.61 |  | 16.44 | 19.74 | 3.68 | 4.97 | 4.80 | 4.18 | 16.81 |  |
|  | p-value, contralateral side |  | <0.001 |  | <0.001 | <0.001 |  | 0.026 | 0.028 | 0.041 | <0.001 |  |

*empty grey fields = no significant difference (p>0.05); R = at rest; WF = Wrinkling of the forehead; CEN = Closing the eyes normally; CEF = Closing the eyes forcefully; WN = Wrinkling of the nose; CMS = Closed mouth smiling; OMS = Open mouth smiling; LP = Lip puckering; BC = Blowing-out the cheeks; S = Snarling; DLL = Depressing lower lip; MF = frontal muscle. medial part; LF = frontal muscle. lateral part; CS = corrugator supercilii muscle; DS = depressor supercilii muscle; OOc = orbibularis oculi muscle; Zyg = zygomatic muscle; LLS = levator labii superioris muscle; Mass = masseter muscle (not innervated by facial nerve. control muscle; OOr = orbicularis oris muscle; DAO = depressor anguli oris muscle; Men = mentalis muscle.

**Supplement Table S4. Kuramoto: Comparison of synkinetic versus contralateral side for the each muscle activity and for each functional facial task separately.***

| **Supplement Table S4.** Kuramoto: Comparison of synkinetic versus contralateral side for the each muscle activity and for each functional facial task separately**.*** | | | | | | | | | | | | |
| --- | --- | --- | --- | --- | --- | --- | --- | --- | --- | --- | --- | --- |
| **Muscle** | **Statistics** | **R** | **WF** | **CEN** | **CEF** | **WN** | **CMS** | **OMS** | **LP** | **BC** | **S** | **DLL** |
| **E9/10** | F-value, T0 |  |  |  | 18.98 | 13.43 |  | 37.06 |  |  | 231.46 |  |
|  | p-value, T0 |  |  |  | <0.001 | <0.001 |  | <0.001 |  |  | <0.001 |  |
|  | F-value, T9 |  |  |  |  | 4.28 |  | 11.45 |  |  | 220.18 |  |
|  | p-value, T9 |  |  |  |  | 0.039 |  | 0.001 |  |  | <0.001 |  |
| **E17/E18** | F-value, T0 |  |  |  | 20.14 | 10.46 | 39.53 | 40.69 |  |  | 6.39 |  |
|  | p-value, T0 |  |  |  | <0.001 | 0.001 | <0.001 | <0.001 |  |  | 0.012 |  |
|  | F-value, T9 |  |  |  | 31.67 | 22.26 | 6.48 | 11.72 |  |  | 8.66 | 12.34 |
|  | p-value, T9 |  |  |  | <0.001 | <0.001 | 0.011 | 0.001 |  |  | 0.003 | <0.001 |
| **E15/16** | F-value, T0 |  | 5.24 | 4.28 | 6.78 | 17.81 |  |  | 10.72 | 6.00 |  | 9.27 |
|  | p-value, T0 |  | 0.022 | 0.039 | 0.009 | <0.001 |  |  | 0.001 | 0.014 |  | 0.002 |
|  | F-value, T9 |  | 7.89 | 4.64 |  | 26.01 | 4.08 | 4.30 | 6.51 | 22.23 |  | 28.79 |
|  | p-value, T9 |  | 0.005 | 0.031 |  | <0.001 | 0.044 | 0.038 | 0.011 | <0.001 |  | <0.001 |
| **E7/8** | F-value, T0 |  |  | 5.33 | 14.27 |  |  |  | 18.15 |  | 9.41 |  |
|  | p-value, T0 |  |  | 0.021 | <0.001 |  |  |  | <0.001 |  | 0.002 |  |
|  | F-value, T9 |  |  | 10.11 | 20.93 | 0.02 | 8.76 | 6.79 | 14.08 |  |  | 4.63 |
|  | p-value, T9 |  |  | 0.001 | <0.001 |  | 0.003 | 0.009 | <0.001 |  |  | 0.031 |
| **E5/6** | F-value, T0 |  |  |  | 321.01 | 26.18 | 13.46 | 11.45 | 11.03 | 23.76 | 18.38 | 12.09 |
|  | p-value, T0 |  |  |  | <0.001 | <0.001 | <0.001 | 0.001 | 0.001 | <0.001 | <0.001 | 0.001 |
|  | F-value, T9 |  |  |  | 305.19 | 35.63 | 8.81 | 12.85 | 4.02 | 22.37 | 18.23 | 15.90 |
|  | p-value, T9 |  |  |  | <0.001 | <0.001 | 0.003 | <0.001 | 0.045 | <0.001 | <0.001 | <0.001 |
| **E13/E14** | F-value, T0 |  | 4.69 |  |  |  |  |  |  |  | 20.33 |  |
|  | p-value, T0 |  | 0.030 |  |  |  |  |  |  |  | <0.001 |  |
|  | F-value, T9 |  |  |  |  |  |  |  |  | 4.03 | 11.93 | 13.27 |
|  | p-value, T9 |  |  |  |  |  |  |  |  | 0.045 | 0.001 | <0.001 |
| **E3/4** | F-value, T0 |  | 42.49 | 7.03 | 24.52 | 10.77 | 33.54 | 42.22 | 48.20 | 36.50 | 66.05 | 32.28 |
|  | p-value, T0 |  | <0.001 | 0.008 | <0.001 | 0.001 | <0.001 | <0.001 | <0.001 | <0.001 | <0.001 | <0.001 |
|  | F-value, T9 |  | 22.91 | 9.14 | 20.58 | 17.39 | 21.16 | 44.48 | 23.16 | 36.66 | 56.25 | 48.38 |
|  | p-value, T9 |  | <0.001 | 0.003 | <0.001 | <0.001 | <0.001 | <0.001 | <0.001 | <0.001 | <0.001 | <0.001 |
| **E1/2** | F-value, T0 |  | 117.20 | 9.46 | 33.64 | 2.95 | 20.65 | 27.31 | 20.14 | 23.37 | 22.82 | 9.70 |
|  | p-value, T0 |  | <0.001 | 0.002 | <0.001 |  | <0.001 | <0.001 | <0.001 | <0.001 | <0.001 | <0.001 |
|  | F-value, T9 |  | 60.28 | 4.91 | 14.60 | 4.26 | 8.93 | 13.87 | 8.90 | 14.34 | 13.47 | 12.42 |
|  | p-value, T9 |  | <0.001 | 0.027 | <0.001 | 0.039 | 0.003 | <0.001 | 0.003 | <0.001 | <0.001 | <0.001 |

*empty grey fields = no significant difference (p>0.05); R = at rest; WF = Wrinkling of the forehead; CEN = Closing the eyes normally; CEF = Closing the eyes forcefully; WN = Wrinkling of the nose; CMS = Closed mouth smiling; OMS = Open mouth smiling; LP = Lip puckering; BC = Blowing-out the cheeks; S = Snarling; DLL = Depressing lower lip; MF = frontal muscle. medial part; LF = frontal muscle. lateral part; CS = corrugator supercilii muscle; DS = depressor supercilii muscle; OOc = orbibularis oculi muscle; Zyg = zygomatic muscle; LLS = levator labii superioris muscle; Mass = masseter muscle (not innervated by facial nerve. control muscle; OOr = orbicularis oris muscle; DAO = depressor anguli oris muscle; Men = mentalis muscle.
